# Discovery and Characterization of a Cryptic Secondary Binding Site in the Molecular Chaperone HSP70

**DOI:** 10.3390/molecules27030817

**Published:** 2022-01-26

**Authors:** Suzanne O’Connor, Yann-Vaï Le Bihan, Isaac M. Westwood, Manjuan Liu, Oi Wei Mak, Gabriel Zazeri, Ana P. R. Povinelli, Alan M. Jones, Rob van Montfort, Jóhannes Reynisson, Ian Collins

**Affiliations:** 1 Cancer Research UK Cancer Therapeutics Unit, The Institute of Cancer Research, London SM2 5NG, UK; oconnosu@tcd.ie (S.O.); Yann-Vai.LeBihan@icr.ac.uk (Y.-V.L.B.); isaacmarkwestwood@gmail.com (I.M.W.); Maggie.Liu@icr.ac.uk (M.L.); Rob.vanMontfort@icr.ac.uk (R.v.M.); 2 School of Pharmacy and Bioengineering, Keele University, Keele ST5 5BG, UK; oiwei.mak@einsteinmed.edu (O.W.M.); j.reynisson@keele.ac.uk (J.R.); 3 Department of Biochemistry, Albert Einstein College of Medicine, Bronx, NY 10461, USA; 4 School of Pharmacy, Institute of Clinical Sciences, College of Medical and Dental Sciences, University of Birmingham, Edgbaston, Birmingham B15 2TT, UK; gabriel.zazeri@unesp.br (G.Z.); ana.povinelli@unesp.br (A.P.R.P.); a.m.jones.2@bham.ac.uk (A.M.J.); 5 Departamento de Física, Instituto de Biociências, Letras e Ciências Exatas (IBILCE), UNESP, Rua Cristovão Colombo 2265, São José do Rio Preto 15054-000, Brazil

**Keywords:** HSP70, cryptic pocket, fragment screen, virtual screen, molecular dynamics

## Abstract

Heat Shock Protein 70s (HSP70s) are key molecular chaperones that are overexpressed in many cancers and often associated with metastasis and poor prognosis. It has proven difficult to develop ATP-competitive, drug-like small molecule inhibitors of HSP70s due to the flexible and hydrophilic nature of the HSP70 ATP-binding site and its high affinity for endogenous nucleotides. The aim of this study was to explore the potential for the inhibition of HSP70 through alternative binding sites using fragment-based approaches. A surface plasmon resonance (SPR) fragment screen designed to detect secondary binding sites in HSP70 led to the identification by X-ray crystallography of a cryptic binding site in the nucleotide-binding domain (NBD) of HSP70 adjacent to the ATP-binding site. Fragment binding was confirmed and characterized as ATP-competitive using SPR and ligand-observed NMR methods. Molecular dynamics simulations were applied to understand the interactions with the protein upon ligand binding, and local secondary structure changes consistent with interconversion between the observed crystal structures with and without the cryptic pocket were detected. A virtual high-throughput screen (vHTS) against the cryptic pocket was conducted, and five compounds with diverse chemical scaffolds were confirmed to bind to HSP70 with micromolar affinity by SPR. These results identified and characterized a new targetable site on HSP70. While targeting HSP70 remains challenging, the new site may provide opportunities to develop allosteric ATP-competitive inhibitors with differentiated physicochemical properties from current series.

## 1. Introduction

Heat Shock Protein 70s (HSP70s) are a family of molecular chaperones with diverse functions, including directly assisting in the correct folding of nascent polypeptides, preventing protein aggregation and aiding the translocation of newly folded proteins to their correct location in the cell [1]. HSP70s have been described as nanomachines that can change the conformation of their substrate polypeptides [2]. This can occur during de novo protein synthesis at the ribosome, with aggregation prone protein intermediates, with stress-denatured proteins or during the assembly and disassembly of protein complexes. HSP70s interact with almost all newly synthesized, partially folded proteins and are able to recognize such a diverse range of proteins by interacting with short motifs of five amino acids, enriched with hydrophobic residues, which are found in practically all polypeptides [3].

HSP70s are implicated in several neurodegenerative diseases involving misfolded proteins, such as Huntington’s, Alzheimer’s and Parkinson’s disease [4]. In cancer, HSP70 function can enhance cell growth, suppress senescence and confer chemotherapeutic resistance. HSP70 expression is associated with metastasis and poor prognosis in many forms of cancer. In particular, HSP70 has been found to be highly elevated in breast, colon, liver, prostate, esophageal and cervical cancers and has been shown to be critical to the growth and survival of many human tumor cell lines [5,6]. The dual silencing of two HSP70 isoforms, HSC70 and HSP72, with siRNA has been shown to cause tumor-specific apoptosis, as well as degradation of the oncogenic proteins CRAF, CDK4 and ERB2 that are clients of the distinct molecular chaperone HSP90. In contrast, apoptosis was not induced in nontumorigenic lines by HSC70/HSP72 siRNA, indicating a potential therapeutic window for an HSP70 inhibitor [7].

The discovery of clinical HSP70 inhibitors has been actively pursued for over a decade but has proven particularly challenging [2]. Studies by X-ray crystallography, NMR and molecular dynamics have shown HSP70 to be a highly flexible protein in which many residues rotate or move substantially [2,8]. In particular, residues in the ATP-binding site can adopt different conformations, meaning the size and shape of the site changes throughout the catalytic cycle and depends on the ligand bound (Supplementary Figure S1). Analyses of the X-ray structure of the nucleotide-binding domain (NBD) of HSC70 show a prevalence of hydrophilic residues in the ATP-binding site (Supplementary Figure S2). Despite the attractiveness of targeting the well-characterized binding site of the endogenous cofactors, designing an inhibitor that targets such polar interactions while maintaining appropriate physicochemical properties for cell permeability and tissue absorption is challenging.

HSP70 has a very high affinity for ADP (K_D_ = 0.5 µM) [9], necessitating orthosteric inhibitors to be potent binders to compete effectively with the endogenous nucleotide cofactors. VER-155008, the most cell active ATP-competitive HSP70 inhibitor described to date (K_D_ HSP70 = 0.5 µM), is based on the adenosine scaffold [9]. The treatment of cancer cells with VER-155008 replicates the phenotype of the genetic knockdown of HSC70/HSP72, and the compound serves as a useful in vitro chemical probe. However, rapid metabolism and clearance in vivo preclude achieving a pharmacologically relevant concentration in tumor tissues.

Compound hit rates in fragment screens can be used to measure the ligandability of a protein of interest [10]. In a fragment screen against the extensively drugged chaperone HSP90, researchers at Vernalis reported a hit rate of 4.4% [11]. In comparison, the hit rate against HSP70 using the same screen was ten-fold lower, at 0.4%, emphasizing the likely difficulty of identifying high-affinity ligands for the NBD of HSP70. However, another fragment screen against HSP70 found compounds that bound to four distinct sites outside of the ATP-binding site [12], suggesting the existence of multiple binding sites in the protein. Overall, six distinct sites for small molecule interactions with full-length HSP70 have been identified or hypothesized thus far, four of which are located in the NBD, including three clustered within or adjacent to the ATP-binding site [2]. Targeting allosteric sites in HSP70 offers the opportunity to overcome the known issues with targeting the orthosteric site described above. We have previously reported a fragment screening approach to HSP70 that identified a series of quinazoline ligands binding in the ATP site [8]. In this study, we describe how further analysis of the output of this SPR screen enabled the discovery of a secondary, cryptic binding site in the NBD of HSP70. We validated and characterized this new binding site using crystallography, biophysical assays and molecular dynamics simulations. We went on to identify novel hit matter targeting HSP70 by virtual screening methods targeting the cryptic site.

## 2. Results

### 2.1. Discovery of a New Cryptic Binding Site in HSP70

A fragment screen was carried out with 1962 fragment compounds using surface plasmon resonance (SPR) to identify the compounds binding to HSP70, as previously described [8]. The full-length HSP72 isoform was found to give erratic and difficult-to-interpret SPR sensorgrams. Therefore, the screen was carried out using a truncated HSC70 nucleotide-binding domain (HSC70-NBD) variant. Comparison of the sequences of HSP72 and HSC70 showed a sequence identity of 85.1% for the full-length proteins, rising to 88.7% when considering the NBDs only (Supplementary Figure S15).

In parallel, we screened for binding to an ATP-binding site mutant S275W HSC70 to differentiate between compounds binding in the primary ATP-binding site from those binding in secondary sites. An overlay of the X-ray structures of wild-type adenosine-bound HSC70-NBD with the apo S275W mutant shows that the adenine ring of adenosine overlaps with the mutant tryptophan residue, indicating that there would be a steric clash between the adenine moiety and the S275W mutant (Supplementary Figure S3). Indeed, introduction of this mutation abolished the binding of adenosine by SPR, as expected [8]. Therefore, hits that bind equally well to both wild-type and S275W mutant HSC70-NBD are likely to bind outside of the binding site of the adenosine base moiety of ATP. We were aware that fragments with weak, non-ATP-competitive binding to both WT and S275W HSC70-NBD could be binding nonspecifically. Therefore, potential dual hit compounds were validated by X-ray crystallography using the HSP72-NBD protein.

**Scheme 1:**
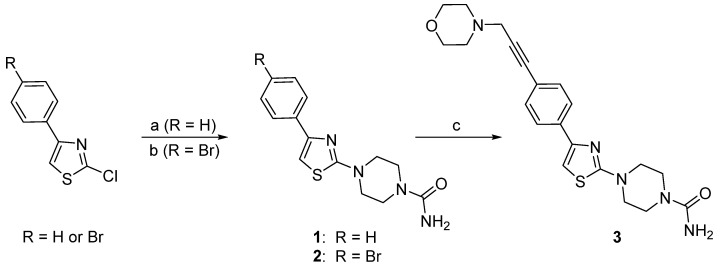
Synthesis of compounds **1** and **3**. (a) Piperazine-1-carboxamide, K_2_CO_3_, DMSO, 130 °C, 7 h (30%). (b) Piperazine-1-carboxamide, ^i^Pr_2_EtN, DMSO, 80 °C, 24 h (23%). (c) 4-(Prop-2-yn-1-yl)morpholine, PdCl_2_(CH_3_CN)_2_, SPhos, K_2_CO_3_, MeCN, 90 °C, 2 h (55%).

Seven fragments with similar SPR responses to wild-type HSC70-NBD and the S275W mutant were assessed by soaking into HSP72-NBD crystals, and a single hit (**1**; Scheme 1) was found to be bound to the protein. The X-ray structure of this commercially available urea **1** bound to HSP72-NBD was solved to 1.79 Å resolution (PDB ID 7Q4R).

**Figure 1:**
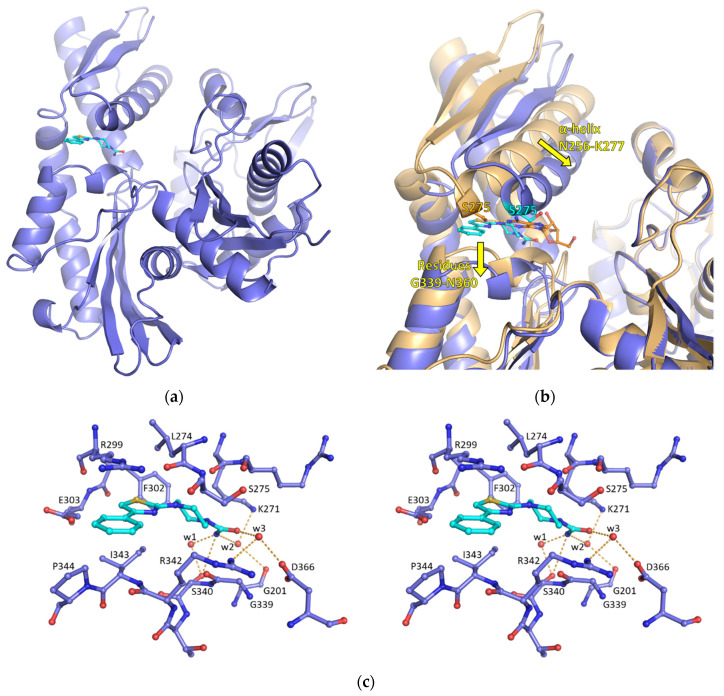
Fragment **1** binds to a previously unknown cryptic secondary binding site in HSP72-NBD. (**a**) X-ray structure of **1** (cyan ball and sticks) bound to HSP72-NBD (blue cartoon; PDB ID: 7Q4R). (**b**) Overlay of the crystal structure of adenosine (orange ball and sticks) bound to HSP72-NBD (light-orange cartoon; PDB ID: 5AQY) with that of **1** bound to HSP72-NBD. The figure was generated by overlaying the carbon alpha atoms of residues 2–174 of HSP72 bound to adenosine or to **1**, a region for which no significant backbone position difference was observed between the 2 structures (RMSD of 0.22 Å). Residue S275 (orange and cyan ball and sticks) is displaced within the ATP-binding site upon the binding of **1** and is no longer positioned to make the anchoring hydrogen bond formed with the N1 of bound adenosine (yellow dashed line). (**c**) Expanded stereo view of the binding site of **1** with key residues labeled. Water molecules are shown as red spheres. Hydrogen bonds are shown as yellow dashed lines.

The structure of **1**-HSP72-NBD revealed the fragment bound in a new secondary binding site located adjacent to the ATP-binding site (Figure 1a). This site has not been described before and is not present in the currently reported apo or nucleotide-bound crystal structures of HSP70 isoforms. The cryptic binding pocket results from movements of the C-terminal subdomain comprising residues D225–L305 relative to the N-terminal subdomains. In particular, the α-helix N256–S277 that lines the nucleotide-binding cleft moves closer to the N-terminal domain than in nucleotide-bound structures, and residues G339–N360 move further away from the helix N256–S277, forming the binding pocket for **1** (Figure 1b). The new pocket consists of an extended narrow tunnel with a mix of hydrophobic and hydrophilic amino acids, in contrast to the larger and more hydrophilic ATP-binding site. The lipophilic 4-phenylthiazole motif of **1** forms mainly hydrophobic interactions with the protein and extends into the canonical nucleotide-binding site, with one single direct hydrogen bond between the compound and the side chain of S340, and additional indirect interactions with G201, K271, S340, R342 and D366 mediated by three water molecules (Figure 1c).

### 2.2. Validation of Fragment Binding through Characterization of Solubilized Analogs in Orthogonal Biophysical Assays

**Figure 2:**
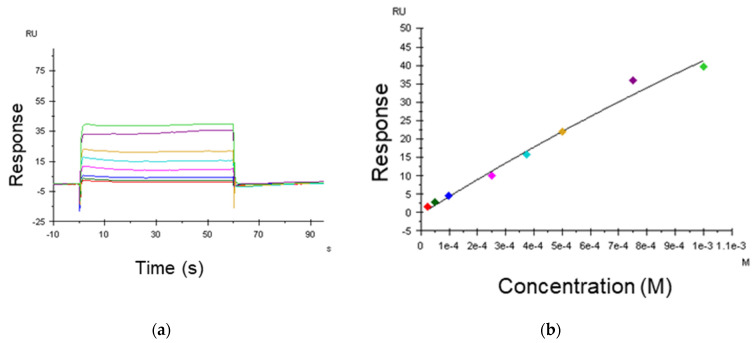
(**a**) Concentration–response SPR sensorgrams for **1** binding to HSC70-NBD and (**b**) corresponding Langmuir-binding isotherm showing non-saturating behavior.

Fragment **1** was resynthesized for detailed study (Scheme 1). Despite the clear evidence of binding to HSP72-NBD by X-ray crystallography, an analysis of the binding of **1** to HSC70-NBD by SPR showed non-saturating behavior, which can indicate a very weakly interacting fragment or nonspecific binding at the high concentrations required to test low-affinity fragments (Figure 2). We hypothesized that the non-saturation of the SPR sensorgrams could be explained by the poor aqueous solubility of **1** at high micromolar to millimolar concentrations (Supplementary Figure S16). We therefore designed a more aqueous soluble version of **1** to robustly confirm fragment binding in biophysical assays and chose to add a protonatable morpholine to the ligand. It was important that the substituent would not interfere with ligand binding, and an examination of the X-ray structure showed the *para* position of the terminal benzene ring in **1** to be solvent exposed and suitable for attachment of the solubilizing group, leading to the design of compound **3** (Scheme 1). The alkynyl substitution was selected to provide a readily synthesized, rigid linker directed away from the protein surface, while the terminal morpholine was chosen as a weakly basic group to aid solubility without perturbing fragment binding (morpholine N, predicted pK_a_ ~ 6.6, ChemDraw 20.1).

Compound **3** was synthesized by the S_N_Ar reaction followed by a Sonogashira coupling (Scheme 1). Low yields were obtained for the conversion of **2** to **3** due to competing product decomposition through cleavage of the urea to form the unsubstituted piperazine.

**Figure 3:**
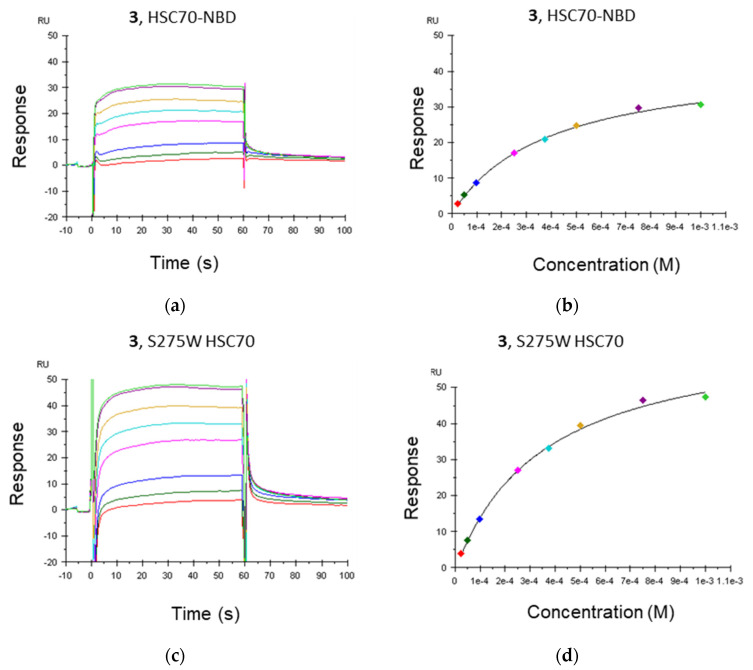
Concentration–response SPR sensorgrams for **3** binding to HSC70-NBD proteins. (**a**) Data for **3** bound to WT HSC70 and (**b**) corresponding Langmuir-binding isotherm. (**c**) Data for **3** bound to S275W HSC70, and (**d**) corresponding Langmuir-binding isotherm.

In contrast to the initial hit **1**, compound **3** gave saturating SPR-binding curves across the concentration range tested, from which the K_D_ could be calculated and giving confidence that the compound bound specifically to the protein in a concentration-dependent manner and with 1:1 stoichiometry (Figure 3 and Table 1). As expected from the binding mode of **1**, analog **3** maintained equivalent affinities for the S275W mutant and for the wild-type HSC70 protein. The calculated ligand efficiency of **3** (LE = 0.16) for binding to the WT protein was low compared to that typically desired in molecules for progression by fragment-based drug design [13].

We hypothesized that **1** and its analog **3** would be ATP- and ADP-competitive by comparing the X-ray structures of adenosine and fragment **1** bound to HSP72-NBD (Figure 1b). Known nucleotide-competitive HSP70 inhibitors formed a key anchoring interaction with S275, mimicking the interaction observed between the N1 ring atom of adenosine and the hydroxyl of S275 (Figure 1b, PDB ID 5AQY) [8]. The binding of **1** in the new secondary site necessitates that the α-helix N256-S277 moves out of position relative to the opposite face of the nucleotide-binding cleft and twists the section from K271–S277, displacing the key S275 residue from the orientation required for a productive interaction with a nucleotide. Thus, when **1** binds to HSP72, the ATP-binding site is disrupted, and we expected that the two binding events would not be possible at the same time. Therefore, ATP or ADP, which have much higher affinities for HSP72, should displace the fragments binding at the secondary site.

**Figure 4:**
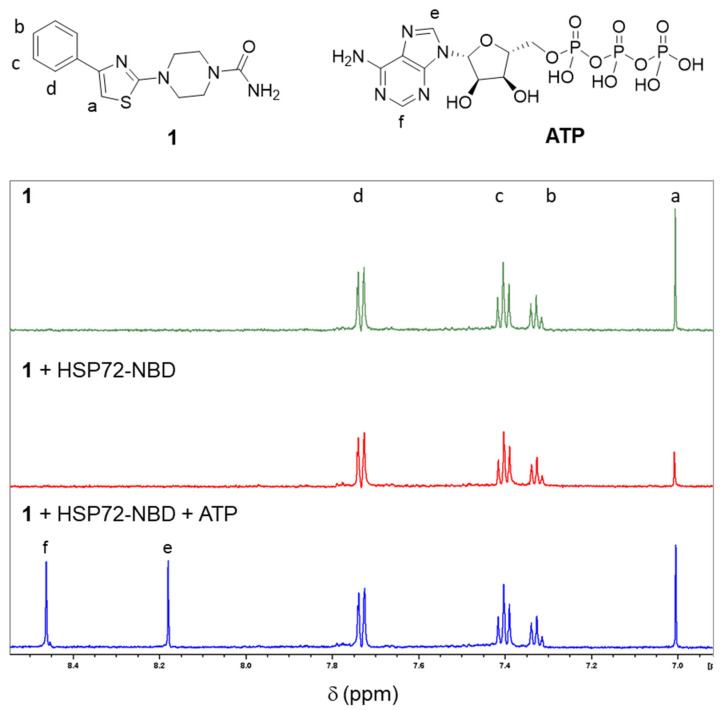
Binding of **1** to HSP72-NBD was detected by CPMG LO-NMR and displaced by the addition of ATP.

To study the binding of **1** and **3** further, and to understand the relationship to nucleotide binding, we used ligand observed (LO) NMR spectroscopic methods. The binding of **1** to HSP72-NBD was assessed using a Carr-Purcell-Meiboom-Gill (CPMG) relaxation edited 1 H NMR experiment (Figure 4). The proton (a) at C5 of the thiazole ring was buried inside the pocket (Figure 1c) and was therefore used to calculate the percentage reduction of the intensity of the ^1^H NMR spectrum signal upon binding. When HSP72-NBD was added to **1**, a significant reduction (58%) of the intensity of the ^1^H NMR signal of the thiazole proton was observed in comparison to the ^1^H NMR spectrum of **1** alone, confirming that the compound was binding to the protein (Figure 4). In contrast, the more solvent-exposed protons on the terminal aromatic ring (b–d) were much less responsive to the change in the environment on protein binding (Table 2). The addition of ATP (e,f) restored the compound signals as close to their original values, confirming the competition between ATP and fragment **1** for binding to HSP72-NBD, as hypothesized. Similar qualitative CPMG experiments were carried out for the substituted analog **3**. This compound also showed binding to HSP72-NBD and displacement by ATP (Supplementary Figure S4 and Table S1).

As the SPR experiments showed signs of nonspecific binding for fragment **1** that could be linked to poor solubility at high concentrations (Figure 2), we attempted to use LO-NMR experiments to differentiate between specific target binding and nonspecific binding due to the formation of microscopic aggregates in aqueous buffers [14]. Compound **1** did not show aggregation in the LO-NMR experiments at the tested concentration of 200 µM. The measurement of the kinetic solubility in pH 7.4 phosphate buffer by an NMR method gave a solubility of 291 µM for **1** (Supplementary Figure S16).

**Figure 5:**
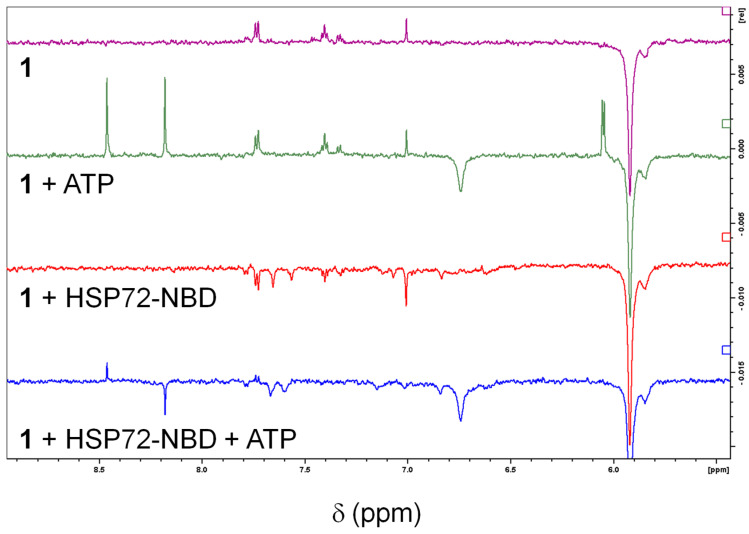
Binding of **1** to HSP72-NBD was detected by waterLOGSY LO-NMR and displaced by the addition of ATP.

A second LO-NMR method, waterLOGSY, was used to observe the binding of **1** to HSP72-NBD. The signs of the observed nuclear Overhauser effects (NOE) of the ligand were inverted upon the addition of HSP72-NBD, indicating the ligand was binding to the protein (Figure 5). Upon the addition of ATP, the inverted signals due to compound **1** NOEs reduced in intensity, again indicating that ATP competes with the fragment for HSP70 binding. Thus, despite the apparent solubility limitations of the original fragment hit **1** in SPR experiments, clear ATP-competitive binding of **1** to HSP70 was demonstrated under nonaggregating conditions by LO-NMR.

### 2.3. Exploring the Interactions of ***1*** with the Secondary Binding Site by Molecular Dynamics

**Figure 6:**
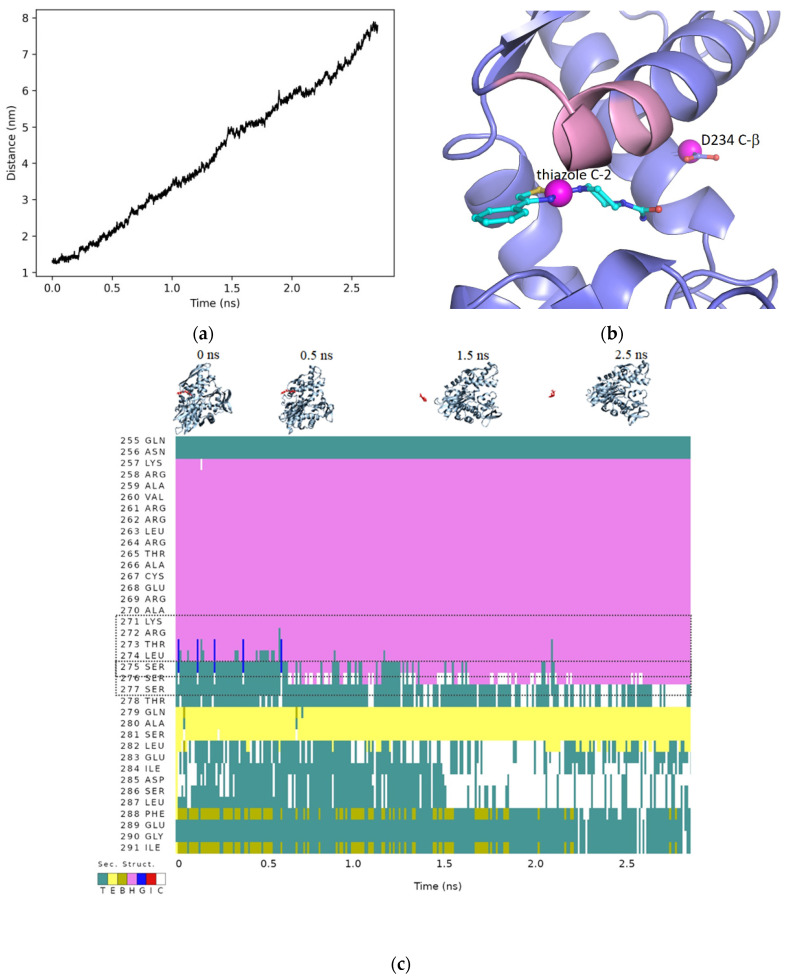
Molecular dynamic simulations on the **1**-HSP72-NBD complex. (**a**) Distance between reference atoms in HSP72 and **1** during the pulling simulation time. (**b**) Structure of **1**-HSP72-NBD (blue cartoon) with the amino acids from K271 to S277 highlighted in pink and **1** in cyan. Large magenta spheres represent the carbon atoms used as reaction coordinates for the pulling simulation. (**c**) Heat map representation of the secondary structures adopted by amino acids Q255–I291 during the pulling of **1**, where T represents the turns, E the β-sheet, B the isolated bridge, H the α-helix, G the 3–10 helix, I the Pi-helix and C the coil structural motifs. The region S271–K277 and residue S275 are highlighted (box).

The binding of **1** to the cryptic secondary site requires rearrangement of the local structure of HSP70 to create the new binding site, with concomitant disruption of the ATP-binding site. A portion of the α-helix that lines the ATP-binding site (residues K271–S277 of HSP72-NBD, PDB ID 5AQY), including the important nucleotide-anchoring residue S275, adopts a more open turn structure. Together with the movement of the whole α-helix relative to the sequence that forms the opposing face of the nucleotide-binding cleft (residues G339-P344 of HSP72-NBD, PDB ID 5AQY), this opens the new pocket (Figure 1b). To understand these local movements, molecular dynamic (MD) simulations of the α-helix K271–S277 were performed in the presence and absence of **1**. To model the dissociation of **1**, the ligand was pulled from the pocket using the distance between the carbon atoms at C-2 of the thiazole moiety of **1** and the β-carbon of D234 as the reaction coordinate (Figure 6). Fragment **1** was pulled linearly without any abrupt movement. The influence of the removal of **1** on the structural conformation of the whole protein was analyzed and showed that the protein remained stable in general, without presenting any extensive global change in the secondary structures during the simulation (Supplementary Figure S5). Local conformational changes of the α-helix K271–S277 were detected. During the initial 0.5 ns of the simulation, while the ligand was inside the pocket, residues L274, S275 and S276 adopted a turn conformation (represented in green in Figure 6c). After 0.5 ns, these three residues more frequently assumed an α-helical conformation (represented in pink in Figure 6c), closer to that observed in the nucleotide-bound structures of HSP72).

**Figure 7:**
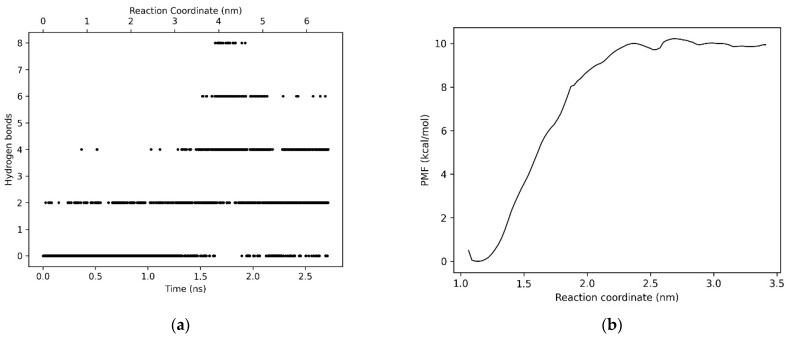
Analysis of the hydrogen bonding interactions of S275 in the MD simulation. (**a**) Number of hydrogen bonds formed between S275 and the residues of HSP72 during the pulling of **1** from the **1**-HSP72-NBD complex. (**b**) Potential of the mean force profile for the pulling of **1** from the HSP72-binding pocket.

To understand the interaction of S275 with adjacent amino acid residues, hydrogen bond formation was monitored (Figure 7a) and showed that, while ligand **1** was placed in the HSP72 pocket (up to 0.5 ns), the number of hydrogen bonds was zero for the majority of this time. After 1.0 ns of pulling, the number of hydrogen bonds increased as S275 began interacting with the adjacent amino acids, contributing to the α-helix stabilization. To obtain the free energy of the system, ΔG, the potential of the mean force (PMF) calculation was performed (Figure 7b). The PMF profile presented a minimum function at approximately 1.15 nm, which was in good agreement with the distance found in the crystallographic data (1.27 nm) for the two carbon atoms used as a reference for the reaction coordinate. From distances greater than 2.5 nm, the fragment **1** lost interactions with the protein and the PMF reached a plateau. The PMF profile led to a ΔG value of −11.2 kcal/mol (−46.8 kJ/mol).

**Figure 8:**
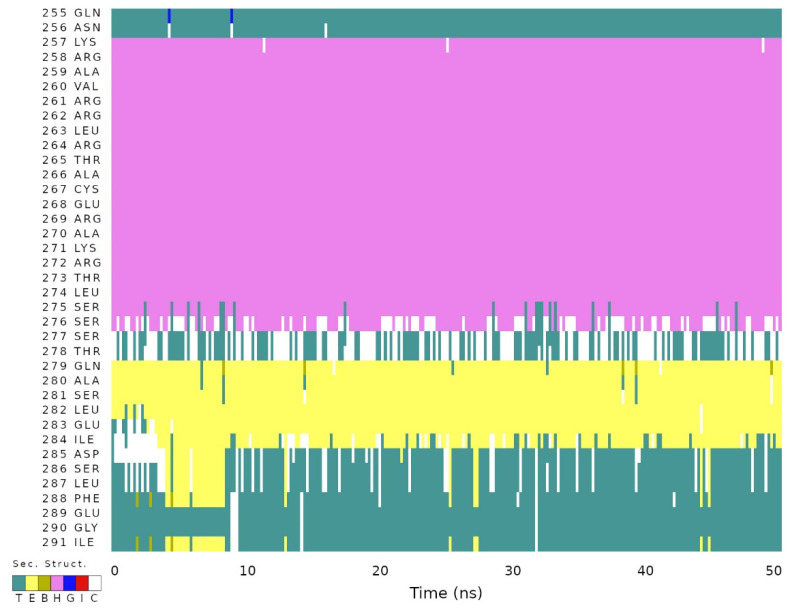
Heat map of the secondary structures adopted by amino acids Q255–I291 HSP72-NBD during the simulation of the HSP72 structure in the absence of a ligand, where T represents the turns, E the β-sheet, B the isolated bridge, H the α-helix, G the 3-10 helix, I the Pi-helix and C the coil structural motifs.

Finally, a MD simulation of the unliganded HSP72-NBD structure was performed to understand the likely frequency with which the cryptic pocket might open spontaneously (Figure 8). The starting structure was constructed by the removal of **1** from the **1**-HSP72-NBD complex, followed by energy minimization and equilibration of the system. Subsequent MD simulation of the protein in the absence of the ligand for 50 ns revealed that residue L274 assumed an α-helix conformation during the full time of the simulation. Similarly, residue S275 assumed an α-helix conformation near continuously, with infrequent fluctuations to the turn conformation associated with the binding of **1**. Thus, the MD simulations overall showed early, localized changes of the HSP72-NBD structure consistent with transition between a conformation containing the cryptic pocket and one suitable for nucleotide binding once ligand **1** is removed. Starting from apo HSP72-NBD in the nucleotide-binding conformation, local movement sufficient to permit the opening of the cryptic pocket was detectable. However, the calculations showed that the α-helix is a conformation of high stability and suggested that conformational change leading to the cryptic pocket occurred infrequently in the absence of ligand **1**.

### 2.4. Virtual High-Thoughput Screening to Identify Alternative Compounds Targeting the Secondary Binding Site

Although the binding of **1** and the solubilized analog **3** to the new secondary site was demonstrated by SPR, LO-NMR and X-ray crystallography, the ligand efficiency of these compounds was low (Table 1), suggesting further progression to more potent compounds would be challenging. In addition, ligands for the site showed competitive binding with nucleotides, and MD simulations suggested spontaneous formation of the secondary binding site to be infrequent. To provide an additional assessment of the tractability of the secondary site towards the discovery of more potent ligands with favorable physicochemical properties, a virtual high-throughput screening using the crystal structure of **1** bound to HSP72 was carried out.

The ChemBridge Diversity library containing 50,004 molecular entities was initially filtered by calculating the Known Drug Indexes (KDIs) for each compound [15]. This method is based on an analysis of the drugs in clinical use, i.e., the statistical distribution of each descriptor is fitted to a Gaussian function and normalized to 1, resulting in a weighted index. Both the summation of the indexes (KDI_2a_) and multiplication (KDI_2b_) methods were used as shown for KDI_2a_ in Equation (1) and for KDI_2b_ in Equation (2).

KDI_2a_ = I_MW_ + I_log P_ + I_HD_ + I_HA_ + I_RB_ + I_PS_ (1)

KDI_2b_ = I_MW_ × I_log P_ × I_HD_ × I_HA_ × I_RB_ × I_PSA_ (2)

A threshold KDI_2a_ value of 5.66 was used with a theoretical maximum of 6 and the average of 4.08 (±1.27) for known drugs. In the case of KDI_2b_, the value of 0.70 was used with a theoretical maximum of 1 and with an average of 0.18 (±0.20) for known drugs, resulting in a compound set containing 10,823 molecular entities with well-balanced physicochemical properties. It was virtually screened against the binding pocket of **1**-HSP72-NBD, with the ligand and water molecules removed, using the four scoring functions available in the GOLD v5.2 software suite: GoldScore (GS), ChemScore (CS), improved Piecewise Linear Potential (ChemPLP) and Astex Statistical Potential (ASP) [16,17,18,19,20]. Redocking of **1** by the protocol used (see the Materials and Methods section) reproduced the experimental binding mode, confirming the reliability of the method used (Supplementary Figure S6).

We applied a virtual screening cascade previously demonstrated to identify active compounds against a variety of targets using different assay formats [21,22,23,24]. From the consensus scoring output of the docking algorithms, molecules with low CS (<25.0), GS (<70.0), ChemPLP (<70.0) and ASP (<27.0), as well as those with limited hydrogen bonding (HB) < 0.2) were eliminated. These numbers were used as compared to the scores for ligands **1** and **3** (CS—30.7/36.0, GS—75.5/91.4, ChemPLP—84.9/102.5 and ASP—34.2/42.8). Only ligands with predicted HB were kept, since HB can contribute to a ligand’s specificity for a given target. The remaining 1173 candidates were screened again with a higher search efficiency. Molecules with low scores CS (<30.0), GS (<75.0), ChemPLP (<80.0), ASP (<33.0) and HB (<1.0) were removed using the same rationale as before, resulting in 166 compounds. These remaining candidates were inspected visually for consensus of the best-predicted conformation of the ligands between the scoring functions, and ligands that showed consistent and plausible conformations were selected, i.e., the ligand conformations were not strained, and the lipophilic moieties did not point into the water environment. Molecules with undesirable functionality linked to cell toxicity and chemical reactivity were also removed from the candidate list [25].

Twenty-four high-ranking compounds from the virtual high-throughput screen were sourced commercially. Compound identity and purity were confirmed by LC-MS analysis (Supplementary Table S2 and Supplementary Figure S14), and the compounds were assayed for binding to HSC70-NBD by SPR, along with two positive controls: adenosine and VER-155008 [9].

Five compounds (**4**–**8**) showed a micromolar affinity for HSC70-NBD by SPR, with saturating binding curves obtained (Table 3 and Supplementary Figure S7). The remaining compounds tested had no binding response or showed super-stoichiometric binding or precipitation that prevented a K_D_ value from being determined. Compounds **4**–**8** are chemically diverse and contain functionalities distinct from the scaffold of **1**. Importantly, vHTS hits **4**–**8** showed similar binding to both WT and S275W HSC70-NBD by SPR, mimicking the behavior of **1** and **3**, consistent with binding outside of the ATP-binding site, as predicted by docking (Supplementary Figures S8–S12). Compounds **4**–**8** had higher molecular weights than fragment **1** (350–386 Da vs. 288 Da) while less than the solubilized analog **3** (411 Da). Some increases in affinity were observed compared to **3**. The ligand efficiencies of the novel hits were improved on in **3**.

## 3. Discussion

Our results demonstrate the effectiveness of a parallel SPR fragment screening strategy using wild-type and ATP site blocked mutant HSP70 NBDs to discover a new ligand-binding site on the HSP70 protein. We reasoned that compounds binding both the wild-type and the ATP site blocked construct could reflect binding to novel sites while also recognizing that such compounds could also be at high risk of promiscuous, nonspecific binding behavior. Parallel screening of a target protein and a control for nonspecific binding has been used to distinguish specific fragment hits [26,27]. Indeed, in our first iteration of this screen, we focused on hits that bound only to the wild-type HSP70 NBD with a competent ATP-binding site [8]. Interestingly, hit **1** showed a non-saturating SPR profile in both proteins studied, more typically associated with nonspecific binding. However, by investigating **1** in X-ray crystallography, we were able to confirm a specific interaction of **1** with the protein. This underlies the importance of applying multiple approaches to understand fragment binding.

The X-ray data for **1** was convincing but not quantitative. We hypothesized that the SPR analysis of **1** could be confounded by limited solubility at high concentrations, and we addressed this by preparing a solubilized derivative **3** designed from the crystal structure of **1** bound to HSP72. This had the dual benefit of enabling the measurement of a ligand-binding affinity for **3** by SPR and of validating the first iteration of a structure-based design from the crystal structure of the novel binding site. Independently, the application of LO-NMR allowed us to confirm the specific binding of both **1** and **3** with an orthogonal technique, where the solubility of **1** did not compromise its performance in the assay. This exemplifies the need to consider the potential confounding effects of low compound solubility relative to the assay conditions and concentration ranges studied. While highly soluble compounds are more likely to exhibit consistent and robust behavior across assay platforms and wide concentration ranges, our findings show that, with appropriate hit modifications and multiple assay techniques, it is possible to validate genuine binding events from less well-behaved chemical starting points [28].

The structure of **1** bound to HSP72-NBD shows the occupation of a cryptic binding pocket. Cryptic sites arise in dynamic regions of a protein and are not observed in known apo protein conformations but become apparent only upon new ligand binding. They may occur by induced fit or by conformational selection when the pocket opens transiently for short periods. Such sites can arise as extensions of an already known pocket or at an entirely new site [29]. In the case of HSP70, it has been challenging to optimize inhibitors that bind the large, highly hydrophilic ATP-binding site and retain optimal physicochemical properties compatible with cell permeability [2]. In contrast, the cryptic pocket has a markedly different shape and distribution of polarity: it is smaller and more enclosed, and while the interior region adjacent to the ATP-binding site involves a network of water molecules and polar amino acids, the main binding tunnel is predominantly hydrophobic. This suggests that substantially different chemical structures would result from optimized inhibitors for this site compared to direct ATP site binders.

Despite their potential in drug discovery, there are limited experimental methods for the direct, detailed structural characterization of the dynamic regions from which cryptic sites arise. As cryptic pockets require ligand binding in order to be identified, the majority have been discovered by serendipity, underlining the importance of experimental screening strategies to uncover new binding sites. Protein-observed NMR has been used to explore the conformational equilibrium between open and closed forms of a cryptic site in an unliganded protein [30]. The use of computational techniques, including molecular dynamics, to search for cryptic pockets has had some success, but there is as yet no general method for predicting new cryptic pockets. Our crystallography data show that the HSP72-NBD pocket that binds **1** opens through specific local movements in a region of the protein with a well-defined secondary structure in the nucleotide-bound form. The local secondary structure changes occur with a realignment of the relative positions of the subregions within the NBD. MD simulations following the de-binding of **1** show movements in the critical helix region K271–S277 that are consistent with the early stages of the trajectory predicted to close the pocket. Importantly, simulation of the protein in the absence of the ligand showed infrequent fluctuations of the local helix to the turn conformation associated with the formation of the cryptic pocket.

Our crystallographic and NMR studies clearly show that the formation of the cryptic pocket, and the binding of ligands to it, is negatively correlated to nucleotide binding at the ATP site of HSP70. As well as changing the ATP site shape, the rearrangement of the helix region K271–S277 to open the cryptic pocket displaces the critical nucleotide-anchoring residue S275. Accordingly, the binding of **1** was competed by ATP in the LO-NMR experiments. As ligands binding to the cryptic pocket will be biochemically competitive with ATP, designing more potent HSP70 inhibitors based on **1** and **3** will encounter a similar challenge in overcoming the high affinity of HSP70 for nucleotides, as is seen for orthosteric inhibitors. The covalent inhibition of HSP70 through the ATP site ligand reaction with an adjacent lysine residue has been demonstrated as an approach to address this challenge [31,32]. More commonly, covalent inhibitors are designed to react with cysteine residues [33]. In the context of the structure of **1**-HSP72-NBD, there are no accessible cysteine residues apparent in the immediate vicinity of the ligand. However, it is interesting to note that the sulfhydryl of residue C306 in HSP72 has been described as susceptible to oxidation, despite its location buried within the core of the subdomain [34]. In **1**-HSP72-NBD, this residue is shielded from the ligand by the sidechain of F302.

Our MD studies indicate that a plausible trajectory exists for local motion in the helix region K271–S277 consistent with opening and closing of the cryptic pocket. Spontaneous movement consistent with the early stages of opening the pocket was seen starting from an apo protein model at a low frequency. The aforementioned competitive mode of action and HSP70’s high affinity for nucleotides, coupled with the stability of the HSP70-NBD structure with the cryptic pocket closed in the MD simulations, imply that reversibly binding ligands targeting the cryptic site will not necessarily offer an advantage in achieving highly potent HSP70 inhibition. The limited accessibility of the site may be reflected in the low ligand efficiencies of the fragment hits.

The effects of HSP70-binding partners on the protein’s conformations will also influence the frequency of the cryptic pocket formation in the cellular environment. For example, nucleotide exchange factors such as BAG1 bind across the distal terminus of the α-helix that begins with the K271–S277 sequence and stabilize a protein conformation with the cryptic pocket closed [35]. While the binding of BAG1 would be predicted to be antagonistic towards the formation of the cryptic pocket, it is also possible that the binding of other partners during the HSP70 client binding and ATP hydrolysis cycle could support the conformational changes needed to open the pocket, presenting a more tractable mechanism for HSP70 binding and inhibition in the cell. Further development to enhance the binding potency of the cryptic site ligands is required to provide reagents suitable to test this hypothesis.

We were able to identify diverse alternative compounds binding HSP72 in the micromolar range by vHTS, using the structure of **1**-HSP72-NBD as a starting point. The ligand efficiencies of the new hits were improved on that of **3**, although remaining lower (<0.3 kJ mol^−1^ HA^−1^) than those typically associated with the rapid progression of a fragment hit to a potent lead within the classical small molecule physicochemical boundaries [13]. Importantly, the physicochemical properties of these compounds are different from some of the nucleotide or nucleotide-mimicking compounds previously identified as the starting points for noncovalent HSP70 inhibitor discovery. While structural similarities of compounds **4**–**8** to compounds **1** and **3** are evident, additional structural biology exploration would inform on the precise binding modes of the new hits.

The HSP70 family is expressed as multiple isoforms in different subcellular locations [2]. Here, we have shown the existence of a cryptic ligand-binding pocket for HSP72-NBD through crystallography and NMR and have correlated this through ligand binding by SPR to HSC70-NBD. Further research is required to understand if the cryptic pocket or a variant is present in the other isoforms of HSP70 and to what extent this pocket may be more or less tractable for drug discovery in other HSP70 isoforms. Equally, improvements in the affinities of the hit matter described here are required to generate compounds that can be used to confirm the functional inhibition of HSP70 and the drugability of the new site. Our identification and characterization of this new secondary binding site and the discovery of fragment hits capable of interacting with it may offer new insights into strategies to inhibit this important molecular chaperone.

## 4. Materials and Methods

### 4.1. Synthetic Chemistry

#### 4.1.1. General Synthetic Method

Anhydrous solvents and reagents were obtained from commercial suppliers and used without further purification. All reactions were performed under nitrogen. Microwave reactions were performed using a Biotage Initiator Microwave Synthesizer (Biotage GB Ltd., Hengoed, UK). Column chromatography was performed on a Biotage SP1 purification system using Biotage SNAP KP-Sil cartridges for normal phase and Biotage SNAP Ultra C18 cartridges for reverse-phase chromatography (Biotage GB Ltd., Hengoed, UK). The mobile phases for reverse-phase chromatography contained 0.1% formic acid. Melting points were determined on a Stanford Research Systems EZ-melt apparatus and uncorrected (Stanford Research Systems, Sunnyvale, CA, USA). Infrared spectra were recorded on a Bruker Alpha-P FTIR spectrometer (Bruker UK Ltd., Coventry, UK). Absorption maxima (V_max_) are quoted in wavenumbers (cm^−1^). ^1^H NMR spectra were recorded on a Bruker AMX500 (500 MHz) (Bruker, Bilerica, MA, USA) spectrometer using an internal deuterium lock. ^13^C NMR spectra were recorded at 126 MHz on a Bruker AMX500 spectrometer using an internal deuterium lock. Chemical shifts were measured in parts per million (ppm) relative to tetramethylsilane (δ = 0) using the following internal references: CDCl_3_ (δ_H_ 7.26; δc 77.2), CD_3_OD (δ_H_ 3.31; δc 49.0) and DMSO-d_6_ (δ_H_ 2.50; δc 39.5). Positive mode LC/MS and HRMS analysis were performed on an Agilent 1200 series HPLC and diode array detector coupled to a 6210 time-of-flight mass spectrometer with a dual multimode APCI/ESI source (Agilent Technologies LDA UK Ltd., Stockport, UK). Analytical separation was carried out at 30 °C on a Merck Chromolith Flash column (RP-18e, 25 × 2 mm) (Merck Life Sciences UK Ltd., Gillingham, UK) using a flow rate of 0.75 mL/min in a 4-min gradient elution with detection at 254 nm. The mobile phase was a mixture of methanol (solvent A) and water (solvent B), both containing formic acid at 0.1%. Gradient elution was as follows: 5:95 (A/B) to 100:0 (A/B) over 2.5 min, 100:0 (A/B) for 1 min and then reversion back to 5:95 (A/B) over 0.1 min and, finally, 5:95 (A/B) for 0.4 min.

#### 4.1.2. 4-(4-Phenylthiazol-2-yl)piperazine-1-carboxamide (**1**)

A mixture of piperazine-1-carboxamide hydrochloride (70.4 mg, 0.425 mmol), 2-bromo-4-phenyl-thiazole (68.0 mg, 0.283 mmol), potassium carbonate (117 mg, 0.850 mmol) and DMSO (1.4 mL) was heated at 130 °C in a microwave reactor for 7 h. Water (20 mL) was added, and the product was extracted with Et_2_O (3 × 20 mL), dried (MgSO_4_), filtered and concentrated. The remaining oil was purified by column chromatography (Biotage SNAP KP-Sil 10 g, eluting with a gradient of 0–7% MeOH in CH_2_Cl_2_). The product was azeotroped with methanol, followed by EtOAc and dried to give **1** as a yellow solid (24.8 mg, 0.086 mmol, 30%). M.p. 131 °C (decomp.); IR (thin film) ν = 3385, 3214, 2858, 1645, 1599, 1531 cm^−1^; ^1^H NMR (500 MHz, methanol-d_4_) δ 7.87–7.81 (m, 2H, ArH), 7.44–7.33 (m, 2H, ArH), 7.31–7.25 (m, 1H, ArH), 7.03 (s, 1H, ArH), 3.63–3.52 (m, 8H, piperazine) ppm; ^13^C NMR (126 MHz, methanol-d_4_) δ 171.2 (C=O), 159.6 (C), 151.6 (C), 135.0 (C), 128.1 (2 × CH), 127.3 (CH), 125.7 (2 × CH), 101.8 (CH), 47.9 (2 x CH2, obscured), 42.9 (2 × CH2); HRMS (ESI+) calculated for C_14_H_17_N_4_OS 289.1118, found 289.1126; HPLC t_R_ = 2.57 min, purity ≥ 95%.

#### 4.1.3. 4-[4-[4-(3-Morpholinoprop-1-ynyl)phenyl]thiazol-2-yl]piperazine-1-carboxamide (**3**)

A mixture of piperazine-1-carboxamide hydrochloride (72.4 mg, 0.437 mmol), 4-(4-bromophenyl)-2-chloro-thiazole (80.0 mg, 0.291 mmol), ^i^Pr_2_EtN (0.100 mL, 0.583 mmol) and DMSO (0.7 mL) was heated in a microwave reactor at 100 °C for 7 h. The reaction mixture was purified by column chromatography (Biotage SNAP-Ultra C18 12 g, eluting with a gradient of 30–100% MeOH in water) to give 4-[4-(4-bromophenyl)thiazol-2-yl]piperazine-1-carboxamide (**2**; 25 mg, 0.067 mmol, 23%) as a white solid. M.p. 151 °C (decomp.); IR (thin film) ν = 3217, 2842, 2121, 2033, 1637, 1587, 1537 cm^−1^; ^1^H NMR (500 MHz, methanol-d4) δ 7.84–7.68 (m, 2H, ArH), 7.61–7.40 (m, 2H, ArH), 7.09 (s, 1H, ArH), 3.66–3.50 (m, 8H, piperazine) ppm; ^13^C NMR (126 MHz, methanol-d_4_) δ 171.2 (C=O), 159.6 (C), 150.3 (C), 134.1 (C), 131.2 (2 × CH), 127.4 (2 × CH), 120.9 (C), 102.5 (CH), 47.9 (2 × CH_2,_ obscured), 42.9 (2 × CH_2_) ppm; HRMS (ESI+) calculated for C_14_H_16_BrN_4_OS 369.0202, found 369.0207; HPLC t_R_ = 2.92 min, purity ≥ 95%.

Bis(acetonitrile)dichloropalladium(II) (5.1 mg, 0.020 mmol) was added to **2** (24.0 mg, 0.065 mmol), SPhos (16.1 mg, 0.039 mmol) and potassium carbonate (36.1 mg, 0.261 mmol). The vial was sealed, and the air was evacuated and replaced with N_2_ three times. Acetonitrile (0.3 mL) and 4-(prop-2-yn-1-yl)morpholine (0.020 mL, 0.131 mmol) were injected. The orange suspension was stirred at 90 °C for 2 h. The reaction mixture was filtered through Celite, concentrated, and purified by column chromatography (Biotage SNAP-Ultra C18 12 g, eluting with a gradient of 10–90% MeOH in water) to give **3** (14.7 mg, 0.036 mmol, 55%) as a brown oil. IR (thin film) ν = 3366, 2924, 2854, 1656, 1601, 1537 cm^−1^; ^1^H NMR (500 MHz, methanol-d_4_) δ 8.17 (s, 2H, NH_2_), 7.91–7.78 (m, 2H, ArH), 7.52–7.37 (m, 2H, ArH), 7.11 (s, 1H, ArH), 3.80–3.75 (m, 4H, morpholine), 3.64 (s, 2H, CH_2_), 3.62-3.55 (m, 8H, piperazine), 2.80–2.73 (m, 4H, morpholine) ppm; ^13^C NMR (126 MHz, methanol-d4) δ 163.9 (C=O), 159.6 (C), 150.71 (C), 135.0 (C), 131.4 (2 × CH), 125.6 (2 × CH), 121.5 (C), 103.0 (CH), 85.9 (C), 83.0 (C), 65.9 (2 × CH_2_), 51.9 (2 × CH_2_), 48.4 (2 × CH_2_), 42.9 (2 × CH_2_), 39.0 (CH_2_) ppm; HRMS (ESI+) calculated for C_21_H_26_N_5_O_2_S 413.1830, found 413.1831; HPLC t_R_ = 2.15 min, purity ≥ 95%.

### 4.2. Biophysical Evaluation of Compounds

#### 4.2.1. SPR Experiments

The HSC70-NBD variants used in SPR were produced and purified as previously described [8]. All surface plasmon resonance (SPR) experiments were carried out on a Biacore T100 enhanced to T200 sensitivity (GE Life Sciences, Amersham, UK), and amine coupling chemistry was used to immobilize the proteins on a research grade CM5 sensor chip. The running buffer was phosphate-buffered saline (10-mM NaHPO_4_-NaH_2_PO_4_, pH 7.4, 2.7-mM KCl and 137-mM NaCl), and the chip’s surface was activated for 10 min using a 1:1 mixture of 100-mM N-hydroxysuccinimide and 400-mM 1-ethyl-3-(3-dimethylaminopropyl)-carbodiimide. Wild-type HSC70-NBD and the S275W mutant proteins were injected for 10 min at a concentration of 2 μM in a 10-mM sodium acetate buffer, pH 5.0 with 100-μM ADP as protection for the active site lysines. Finally, the surface was blocked via an injection of 1 M ethanolamine, pH 8.5 for 7 min. The flow rate was maintained at 10 μL/min for all the above procedures. On average, ~12,000 response units (RU) of the wild-type and the mutant proteins were immobilized on the chip. Flow cell one was left unmodified as the reference surface. Following protein immobilization, the running buffer was changed to phosphate-buffered saline containing 0.05% Tween20 (*v/v*) and 5% DMSO.

To determine binding constants (K_D_) for the compounds, all liquid handling was carried out using an ECHO 550 acoustic liquid dispenser (Labcyte, Dublin, Ireland), and compounds were added to 384-well polypropylene V-bottomed plates (Greiner, Stonehouse, UK), which were used as sample plates for the SPR experiments. Fresh 100-mM DMSO stocks of each compound were prepared and used to generate eight-point concentration responses ranging from 25 μM to 1000 μM or from 6.25 μM to 500 μM. For adenosine, the concentration range was 50 μM–2000 μM from a 200-mM stock in DMSO, and for VER155008, a stock solution of 0.25 mM in DMSO was prepared with a concentration range of 0.0625–2.5 μM. The buffer mix was made compatible with the Biacore running buffer, and the experiments were performed at a flow rate of 30 μL/min, a sample injection time of 60 s and a dissociation time of 200 s. The CM5 surface was not regenerated between sample injections. Binding constants (K_D_) were calculated from the DMSO-corrected and background-normalized binding curves generated from the sensorgrams under equilibrium conditions using the 1:1 binding model in Biacore software version 2 (GE Life Sciences, Amersham, UK).

#### 4.2.2. Ligand Observed NMR Experiments

To confirm the binding of compounds to HSP72-NBD, a series of CPMG and WaterLOGSY experiments were performed. Compounds were assayed at 200 μM in the presence or absence of 10-μM HSP72-NBD. Two hundred micrometers of ATP were also included in the competition experiments. The total assay volume was 200 μL, and the experiments were performed in 3-mm NMR tubes. The buffer was 25-mM Tris, pH 7.5, 50-mM NaCl, 10% D_2_O and 100 μM of DTT in deionized water. NMR experiments were conducted at a ^1^H frequency of 600 MHz using a Bruker Avance 600 spectrometer (Bruker, Bilerica, MA, USA) equipped with a 5-mm TCI Cryo-probe. All data were acquired and processed using Topspin (Bruker, Bilerica, MA, USA) and MNova (Mestrelab Research SL, Santiago de Compostela, Spain). The relaxation-edited 1H-NMR spectrum was acquired at 298 K using the CPMG sequence with a spin-lock time of 600 ms. The water signal was suppressed using pre-saturation during the relaxation delay (2 s) and by using the Watergate sequence subsequent to the CPMG sequence. For each spectrum, 64 transients were acquired.

#### 4.2.3. Determination of the Solubility of **1** by Quantitative NMR

Nine microliters of a 10-mM DMSO solution of **1** was pipetted into one well of a 384-deep well plate (Greiner, part-no. 781270). one hundred and seventy-one microliters of aqueous buffer (137-mM NaCl, 2.7-mM KCl and 10-mM phosphate buffer solution (pH 7.4)) were pipetted into the same well and mixed 3 times by pipette to give a 500-µM solution of **1** with 5% DMSO. The plate was centrifuged (1000 rpm for 1 min, Eppendorf 5810C) and then sealed and incubated at room temperature for 20 h without shaking. The plate was centrifuged (1000 rpm for 1 min, Eppendorf 5810R), and 165 µL of the supernatant was transferred to a 3-mm NMR tube (Bruker, Part No. Z112272) using a liquid handler SamplePro Tube SJ S (Bruker, Bilerica, MA, USA)). The concentration of **1** was measured by quantitative ^1^H-NMR using a single external standard (200-µM caffeine (Sigma C1778, Merck Life Science UK, Gillingham, UK)) in PBS (pH 7.4) with 1% DMSO-d6). NMR data was collected on a Bruker Avance Neo 600 spectrometer equipped with a 5-mm TCI Cryo-probe. The ^1^H spectrum was referenced to the internal deuterated solvent. The operating frequency for ^1^H was 600 MHz. All NMR data were acquired at 298 K. Data were acquired and processed using Topspin 4.0 (Bruker, Bilerica, MA, USA). The quantitative ^1^H-NMR spectrum was acquired using a Bruker standard 1D lc1pngppsf2 pulse sequence with 32 scans. The sweep width was 6.2 ppm with O1P set to 8.8 ppm, and the FID contained 16k time domain data points. The relaxation delay was set to 20 s, and the water signal was suppressed. The quantitative 1H-NMR data analysis was carried out in Mnova 14.1 (Mestrelab Research SL, Santiago de Compostela, Spain).

### 4.3. Protein Crystallography

#### 4.3.1. HSP72-NBD Expression

As previously described [31], the coding sequence for residues 1–380 of HSP72 was inserted into pGEX-6P-1 plasmid to generate a GST-HSP72-NBD fusion construct, where the glutathione-S-transferase tag could be cleaved with PreScission protease. BL21-AI *E. coli* cells were transformed with this plasmid and grown in LB media supplemented with 100-mg/L ampicillin at 37 °C until an OD_600 nm_ of 0.6 was reached. Protein expression was then induced by addition of 0.2-mM IPTG and 0.2% arabinose, and expression was carried out at 18 °C for 18 h. Cells were harvested by centrifugation (5500× *g* for 30 min at 4 °C) and stored at −80 °C.

#### 4.3.2. HSP72-NBD Purification

Cells were resuspended in a lysis buffer composed of 25-mM Tris, pH 7.5, 50-mM NaCl, 2-mM EDTA, 1-mM DTT and 5% glycerol, supplemented with 1 × cOmplete ULTRA protease inhibitors, 12.5-U/mL benzonaze, 1-mM MgCl_2_ and 1-mg/mL lysozyme. Cells were lysed by sonication followed by centrifugation at 21,000× *g* for 40 min at 4 °C. The supernatant was loaded onto a 5-mL GSTrap FF column followed by a wash in lysis buffer, a second wash in lysis buffer at 500-mM NaCl to eliminate bound nucleotides and an elution with lysis buffer supplemented with 20-mM L-glutathione. Fractions containing GST-HSP72-NBD were pooled, followed by the addition of 5-U/mg PreScission protease and incubation at 4 °C for 4 h. Subsequently, the sample was further purified using a HiLoad 26/60 Superdex 200-pg size exclusion column equilibrated in 25-mM Tris, pH 7.5, 50-mM NaCl, 1-mM DTT and 5% glycerol, with the output connected to a GSTrap FF column to eliminate the GST tag. Fractions containing HSP72-NBD were pooled and further purified using a 6-mL Resource Q anion exchange column equilibrated in 25-mM Tris, pH 7.5, 2-mM EDTA, 1-mM DTT and 5% glycerol. The protein of interest was collected in the flow-through and contaminants eluted by applying a buffer at 1-M NaCl. The pooled fractions were concentrated and loaded onto a HiLoad 26/60 Superdex 200-pg gel filtration column equilibrated in a buffer containing 25-mM Tris, pH 7.5, 50-mM NaCl, 1-mM DTT and 5% glycerol. The protein was assessed for purity and molar mass by SDS-PAGE and high-resolution mass spectrometry, respectively. The final sample was concentrated to 10 mg/mL and stored at −80 °C.

#### 4.3.3. HSP72-NBD Crystallization

Purified HSP72-NBD protein was thawed, buffer exchanged into fresh 100-mM HEPES, pH 7.5 and concentrated to 6 mg/mL using a centrifugal concentrator with a 10-kDa molecular weight cut-off. The protein was then incubated with 5-mM adenosine for 30 min on ice prior to crystallization. HSP72-NBD/adenosine cocrystals were grown at 18 °C in sitting drops by mixing 0.5 μL of protein solution and 0.5 μL of precipitant solution consisting of 18–28% PEG 3350, 0.1-M HEPES, pH 7.5, 2-mM MgCl_2_ and 2-mM NaH_2_PO_4_. The crystals typically grew in 2 days. To back-soak adenosine and soak in the compound of interest **1**, crystals were transferred into fresh solutions containing 20% PEG 3350, 0.1-M HEPES, pH 7.5, 2-mM MgCl_2_, 2-mM NaH_2_PO_4_, 50-mM compound **1** and 20% DMSO and incubated for 16 h at 18 °C prior to cryoprotection in paratone-N and flash cooling in liquid nitrogen.

#### 4.3.4. Crystallographic Data Collection, Processing and Refinement

X-ray diffraction data were collected on beamline I24 at Diamond Light Source, Harwell campus, Oxfordshire, UK. Data were integrated with XDS [36] and scaled and merged with AIMLESS [37]. The crystal belonged to the space group P212121 and diffracted to 1.79 Å resolution. The structure was solved by molecular replacement using PHASER [38]. The structure was manually corrected and rebuilt in COOT [39] and refined with BUSTER [40] in iterative cycles. Ligand restraints were generated with GRADE [41] and MOGUL [42]. The quality of the structure was assessed with MOLPROBITY [43,44]. The data collection and refinement statistics are presented in Supplementary Table S3.

### 4.4. Molecular Dynamics Simulations

MD simulation of HSP72-NBD was performed with GROMOS54a7 force field [45] by Gromacs v.5.1.4 [46]. The HSP72-NBD was placed in a cubic box, solvated with the simple point charge water (SPC) [47] and neutralized with NaCl. The energy minimization was performed with steepest descent with tolerance of 10 kJ/mol. The first step of equilibration was performed in the NVT ensemble for 100 ps. The system was coupled to the V-rescale thermostat [48] at 298 K. All bonds were constrained with LINCS algorithm [49], the cutoff for short-range non-bonded interactions was set 1.4 nm and long-range electrostatics was calculated using the Particle Mesh Ewald (PME) algorithm [50]. The second step of equilibration was performed in the NPT ensemble for 100 ps. The system was coupled to Parrinello-Raman barostat [51] to isotropically regulate the pressure. For data collection, the restraints were turned off and the system was simulated along 50 ns with steps of 2 fs.

The same protocol of energy minimization and equilibration was followed for the simulations of 1-HSP72-NBD complex. The topology of **1** was calculated by ATB webserver [52]. The pulling simulation was performed without restraints to allow the protein conformational changes. **1** was pulled away from HSP72-NBD pocket in Z direction over 3 ns, using a spring constant of 700 kJ/mol^−1^nm^−2^ and a pull rate of 2 nm/ns. The distance between the carbon atoms at C-2 of the thiazole moiety of **1** and β-carbon of D234 (Figure 7) was used as the reaction coordinate (ξ). The secondary structures along the pulling simulation were calculated by VMD [53]. The hydrogen bond number was calculated with *gmx hbond* using cutoff radius and angle as 0.35 nm and 30 degrees, respectively. For umbrella sampling, 17 frames from the pulling simulation were extracted and then every frame was subjected to 10 ns of molecular dynamics. The PMF along the reaction coordinate was calculated with *gmx wham* [54].

### 4.5. Docking and Virtual Screening Experiments

The compounds were docked to the fragment-bound (**1**) HSP72-NBD PDB structure (PDB ID: 7Q4R). The Scigress version FJ 2.6 program (Scigress Explorer Ultra Version 2.6. Fujitsu Limited: 2000–2007) was used to prepare the crystal structure for docking, i.e., hydrogen atoms were added, the crystallised ligand **1** was removed as well as crystallographic water molecules. The center of the binding pocket was defined as the position of the nitrogen atom in the thiazole moiety of **1** (*x* = −23.075, *y* = 10.028, *z* = −15.986) with a pocket radius of 10 Å. For the initial screen 30% search efficiency was used (virtual screen) with 10 generic algorithm (GA) runs per compound. For the second phase 100% efficiency was used in conjunction with 50 docking runs. The GoldScore (GS) [16], ChemScore (CS) [17,18] improved Piecewise Linear Potential (ChemPLP) [19] and Astex Statistical Potential (ASP) [20] scoring functions were implemented to validate the predicted binding modes and relative energies of the ligands using the GOLD v5.2 software suite. The virtual high throughput screen was conducted with the ChemBridge Diversity Library [55]. The QikProp 3.2 software package (QikProp version 3.2: Schrödinger, New York, NY, USA, 2009) was used to calculate the molecular descriptors of the molecules. The reliability of this approach is established for the calculated descriptors [56]. The Known Drug Indexes (KDIs) were calculated from the molecular descriptors as described by Eurtivong and Reynisson [15].

## Figures and Tables

**Table 1:** Calculated K_D_ and ligand efficiency values for **3** binding to HSC70 proteins.

Compound	K_D_ (μM) ^1^ HSC70-NBD	Ligand Efficiency (kJ mol^−1^ HA^−1^) ^2^	K_D_ (μM) ^1^ S275W HSC70
**3**	388	0.16	352

^1^ Single determination, calculated from the data shown in Figure 3. ^2^ Calculated for binding to WT HSC70-NBD.

**Table 2:** Observed changes in NMR signal intensities for **1** on the addition of HSP72-NBD and ATP.

	Reduction in ^1^H NMR Peak Intensity Relative to 1 Alone ^1^			
Conditions	Peak a	Peak b	Peak c	Peak d
**1** + HSP72-NBD	58%	7%	14%	13%
**1** + HSP72-NBD + ATP	8%	12%	2%	2%

^1^ Quantified from the data shown in Figure 4 using MestreNova.

**Table 3:** Structures and affinities determined by the SPR of vHTS hits for HSC70-NBD.

vHTS Hit	HSC70-NBD SPR K_D_ (μM) ^1^	Ligand Efficiency (kJ mol^−1^ HA^−1^)	HSC70-NBD S275W SPR K_D_ (μM) ^2^
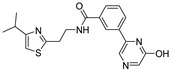 **4**	406 (±110)	0.18	385 (±97)
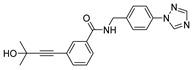 **5**	295 (±59) ^2^	0.19	176 (±88)
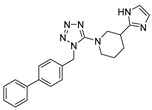 **6**	13 (±1) ^2^	0.24	12 ^3^
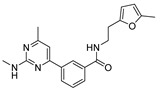 **7**	134 (±69)	0.21	159 (±55)
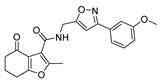 **8**	99 (±14)	0.20	86 (±12)

^1^ Mean (±SD) for *n* = 3 determinations. Representative data are shown in Supplementary Figure S7. ^2^ *n* = 2 determinations. ^3^ Single determination.

## Data Availability

Atomic coordinates and structure factors for the crystal structure of **1**-HSP71-NBD have been deposited with the RCSB Protein Databank, accession code 7Q4R, and will be publicly released upon article publication.

## Supplementary Materials

The following are available online, Figure S1: HSP70 ATP-binding site structures, Figure S2: ATP interactions with HSP70, Figure S3: ATP site structure in S275W HSC70, Figure S4: Binding of **3** to HSP72-NBD, Figure S5: Protein structural changes during ligand pulling MD simulation, Figure S6: Validation of HSP70 docking protocol, Figure S7: SPR profiles of vHTS hits binding HSC70-NBD, Figure S8: The docked pose of compound **4** in the cryptic pocket of HSP70 as predicted by the ASP scoring function, Figure S9: The docked pose of compound 5 in the cryptic pocket of HSP70 as predicted by the ASP scoring function, Figure S10: The docked pose of compound **6** in the cryptic pocket of HSP70 as predicted by the ASP scoring function, Figure S11: The docked pose of compound **7** in the cryptic pocket of HSP70 as predicted by the ASP scoring function, Figure S12: The docked pose of compound **8** in the cryptic pocket of HSP70 as predicted by the ASP scoring function, Figure S13: 1H and ^13^C NMR of compounds **1**, **2** and **3**, Figure S14: LC-MS of compounds **1**–**8**, Figure S15: Sequence alignment of the nucleotide-binding domains of human HSP72 and HSC70 proteins, Figure S16: Determination of buffer solubility of **1** by quantitative NMR, Table S1: Observed changes in NMR signal intensities for **3** on addition of HSP72-NBD and ATP, Table S2: LC-MS data to confirm the identity and purity of vHTS hits **4**–**8**, Table S3: Crystallographic data collection and refinement statistics for **1**-HSP72-NBD (7Q4R).

## Sample Availability

Samples of the compounds **1** and **3** are available from the authors.
