# Supplementary material for: Discovery and Characterization of a Cryptic Secondary Binding Site in the Molecular Chaperone HSP70
Source: Molecules. 2022 Jan 26;27(3):817. doi: 10.3390/molecules27030817 (PMC8839746; doi:10.3390/molecules27030817)
Supplement: Supplementary file 1 [file molecules-27-00817-s001.zip › molecules-1535967-supplementary.pdf]

Discovery and characterization of a cryptic secondary binding site in the molecular chaperone HSP70

Suzanne O'Connor [1], Yann-Vai Le Bihan [1], Isaac M. Westwood [1], Manchin Liu [1], Oi Wei Mak [3], Gabriel Zazeri [4,5], Ana Povinelli [4,5], Alan M. Jones [4], Rob van Montfort [1], Jóhannes Reynisson [2] and Ian Collins [1,*]

[1] Cancer Research UK Cancer Therapeutics Unit, The Institute of Cancer Research, London, SM2 5NG, UK
[2] School of Pharmacy and Bioengineering, Keele University, Keele, ST5 5BG, UK
[3] Department of Biochemistry, Albert Einstein College of Medicine, Bronx, NY 10461, USA
[4] Institute of Clinical Sciences, University of Birmingham, Edgbaston, Birmingham, B15 2TT, UK
[5] Departamento de Física, Instituto de Biociências, Letras e Ciências Exatas (IBILCE), UNESP, Rua Cristovão Colombo 2265, 15054-000 São José do Rio Preto, Brazil
* Correspondence: ian.collins@icr.ac.uk

Supplementary Materials

Figure S1: HSP70 ATP binding site structures.

Figure S2: ATP interactions with HSP70.

Figure S3: ATP site structure in S275W HSC70.

Figure S4: Binding of **3** to HSP72-NBD.

Figure S5: Protein structural changes during ligand pulling MD simulation**.**

Figure S6: Validation of HSP70 docking protocol.

Figure S7: SPR profiles of vHTS hits binding HSC70-NBD.

Figure S8: The docked pose of compound **4** in the cryptic pocket of HSP70 as predicted by the ASP scoring function.

Figure S9: The docked pose of compound **5** in the cryptic pocket of HSP70 as predicted by the ASP scoring function.

Figure S10: The docked pose of compound **6** in the cryptic pocket of HSP70 as predicted by the ASP scoring function.

Figure S11: The docked pose of compound **7** in the cryptic pocket of HSP70 as predicted by the ASP scoring function.

Figure S12: The docked pose of compound **8** in the cryptic pocket of HSP70 as predicted by the ASP scoring function.

Figure S13: 1H and 13 C NMR spectra for compounds **1**, **2** and **3**.

Figure S14: LC-MS spectra for compounds **1** – **8**.

Figure S15: Sequence alignment of the nucleotide binding domains of human HSP72 and HSC70 proteins.

Figure S16: Determination of buffer solubility of **1** by quantitative NMR.

Table S1: Observed changes in NMR signal intensities for **3** on addition of HSP72-NBD and ATP.

Table S2: LC-MS data to confirm the identity and purity of vHTS hits **4** – **8**.

Table S3: Crystallographic data collection and refinement statistics for **1**-HSP72-NBD.

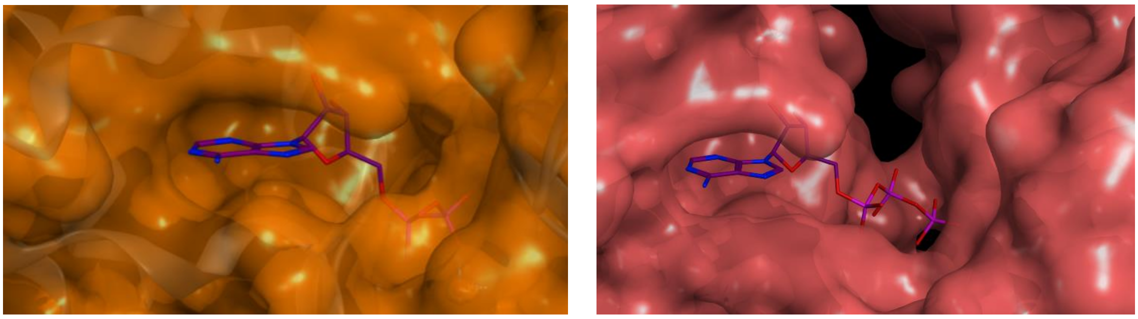

(**a**) (**b**)

Supplementary Figure S1. HSP70 ATP binding site structures**.** The HSP70 ATP-binding site is highly flexible with the size and shape dependent on nucleotide binding and association with nucleotide exchange factors such as Bag1. (**a**) Structure of HSC70-NBD in complex with ADP (PDB ID: 4H5T). (**b**) Structure of HSC70/Bag1 in complex with ATP (PDB ID: 2KHO).

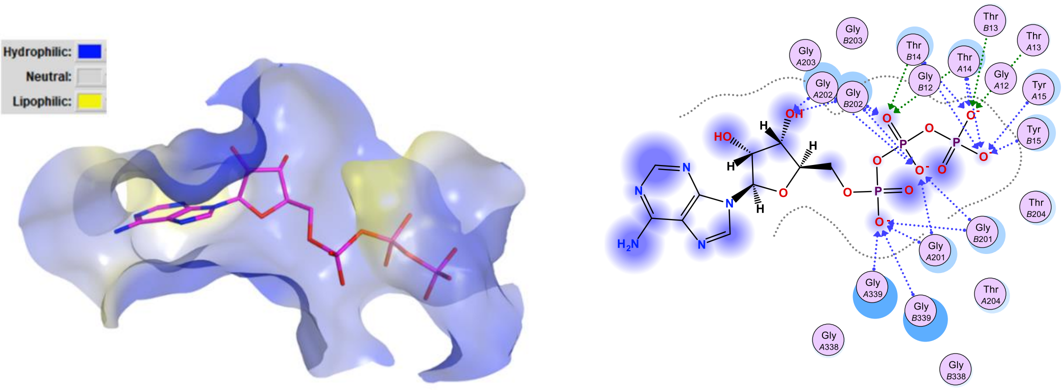

(**a**) (**b**)

Supplementary Figure S2. ATP interactions with HSC70. (**a**) Representation of the surface of the ATP-binding site of HSC70/Bag 1 in complex with ATP (sticks; PDB ID: 3FZF) showing the prevalence of hydrophilic (blue) in comparison to lipophilic (yellow) and neutral (grey) residues on the surface. (**b**) Ligand interaction diagram showing the binding of the β– and γ-phosphates of ATP stabilized by interactions with polar amino acids, water and cations in the binding site.

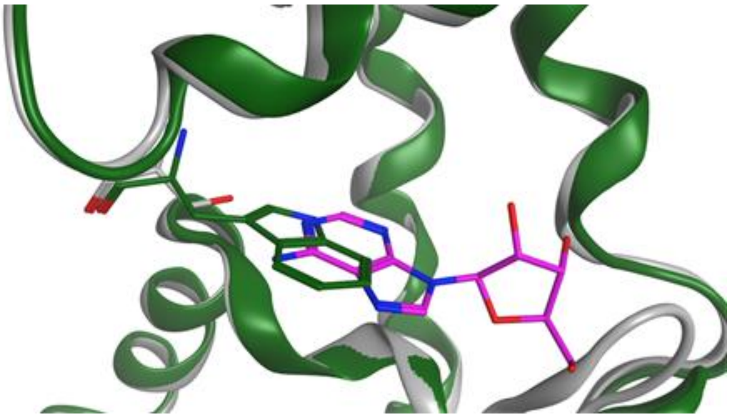

Supplementary Figure S3. ATP site structure in S275W HSC70**.** Overlay of wild type HSC70-NBD bound to adenosine (PDB ID 5AQF; grey) with the S275W mutant HSC70-NBD (PDB ID 5AQL; green). The large indole group of the tryptophan (green sticks) occupies the space where the adenine ring of adenosine (magenta sticks) would normally bind.

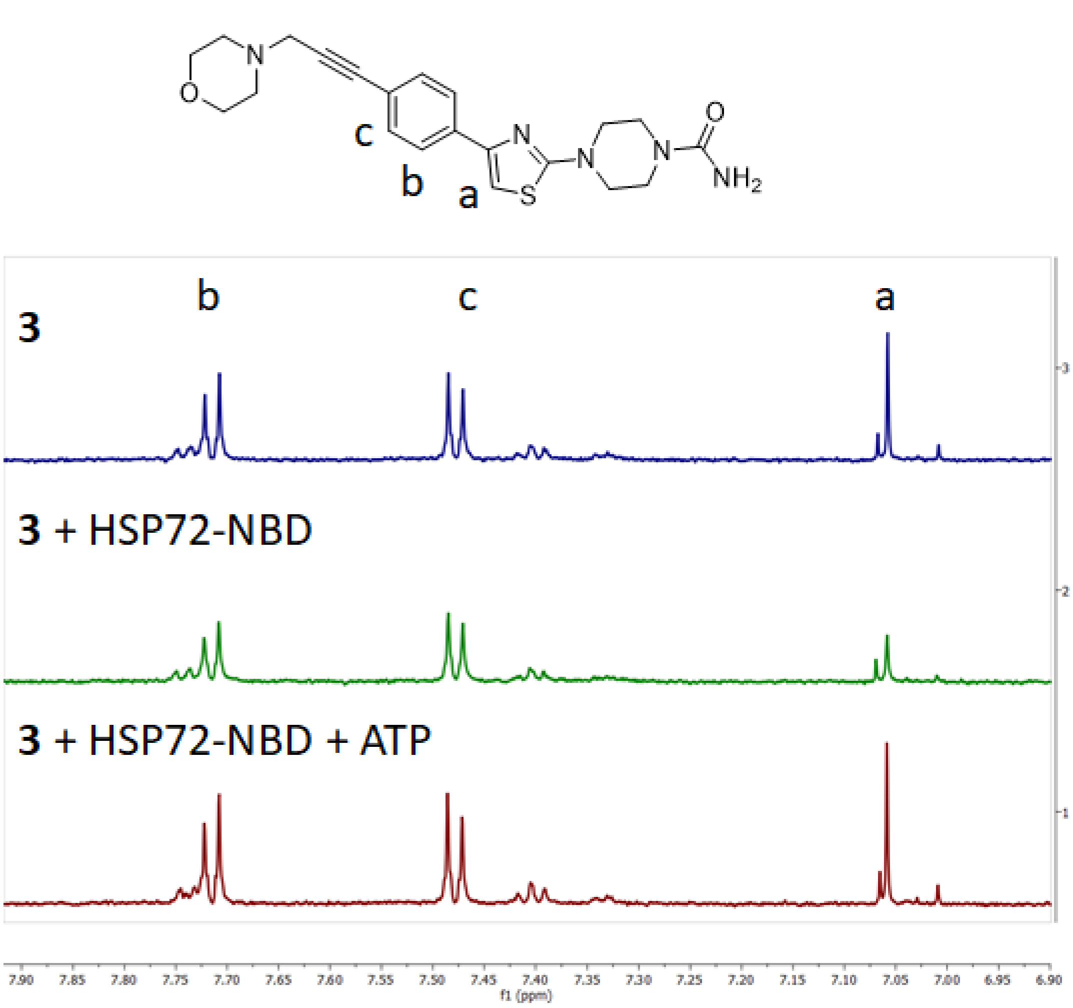

Supplementary Figure S4. Binding of **3** to HSP72-NBD was detected by CPMG LO-NMR and displaced by the addition of ATP.

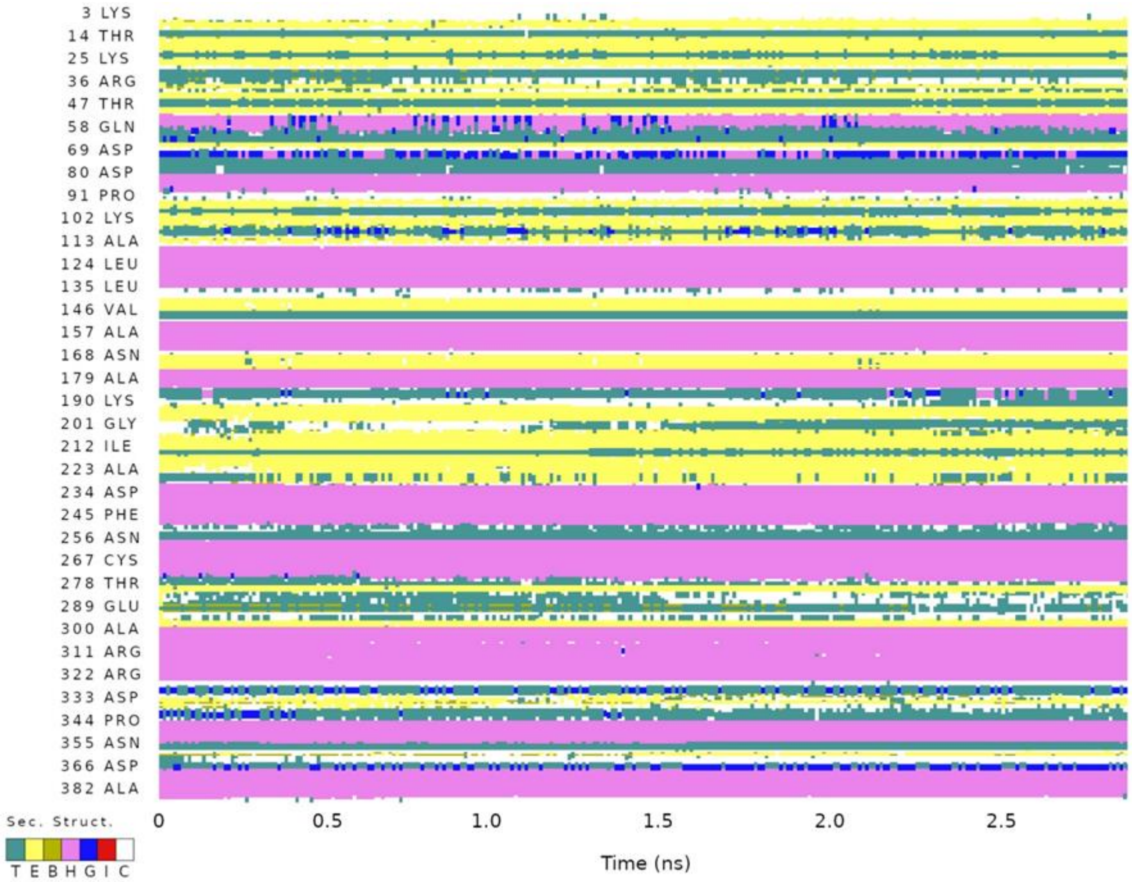

Supplementary Figure S5. Protein structural changes during ligand pulling MD simulation**.** Heat map representation of HSP72-NBD secondary structures showing they are broadly maintained during the MD pulling experiment to remove **1** from the secondary binding site; where T represents the turns, E the β-sheet, B the isolated bridge, H the α-helix, G the 3-10 helix, I the Pi-helix and C the coil structures.

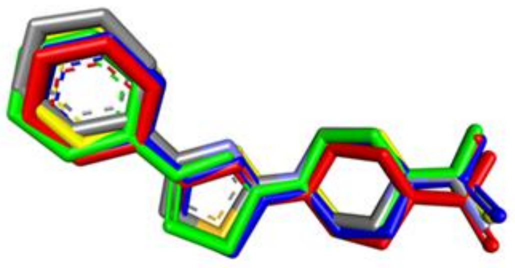

Supplementary Figure S6. Validation of HSP70 docking protocol. Overlays of the docked binding poses of **1** with the crystallographic data for **1**-HSP72-NB (PDB ID: 7Q4R). The crystal structure conformation of **1** is colored by element, the docked conformations are colored as: ChemPLP, blue; CS, red; GS, yellow, and ASP, green. The root-mean-square deviation (RMSD) between the heavy atoms of **1** in the crystal structure conformation and the predicted binding poses are: ChemPLP – 0.8422 Å, CS – 0.9190 Å, GS – 0.9602 Å and ASP – 0.7938 Å. RMSD values < 1 indicate excellent reproduction of the experimental situation.

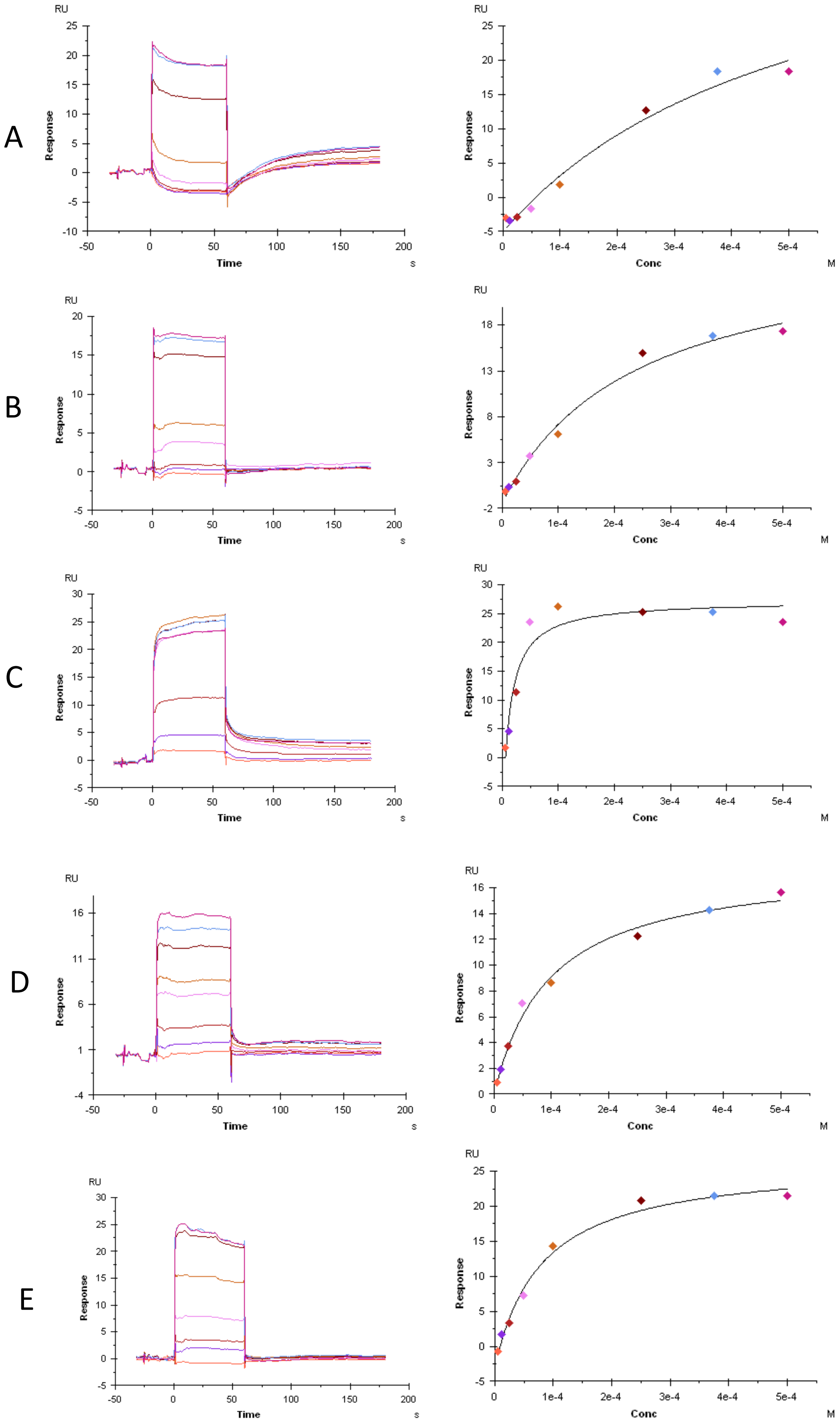

A
B
C
D
E
RU
Response
Time
s
Conc
M
-50
0
50
100
150
200
1e-4
2e-4
3e-4
4e-4
5e-4

F

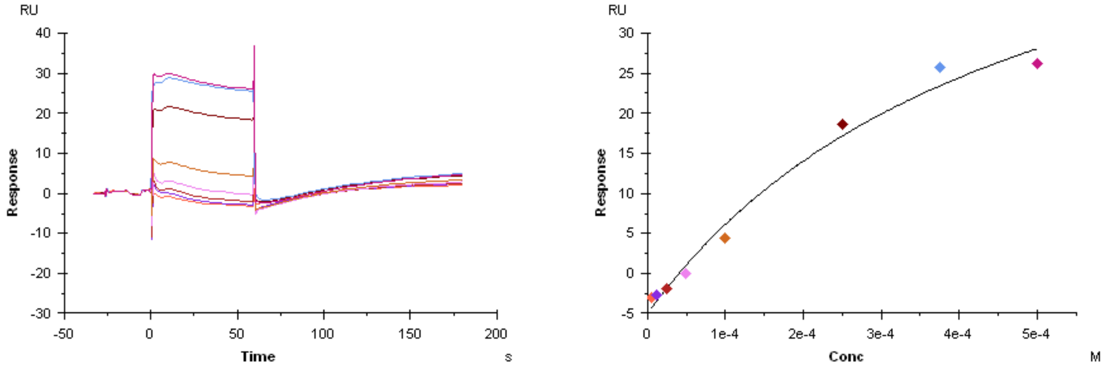
RU
Response
Time
s
RU
Response
Conc
M

G

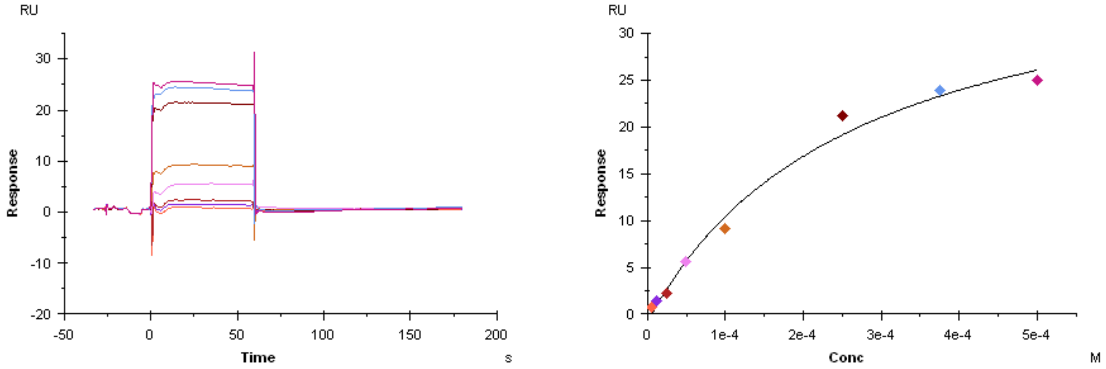
RU
Response
Time
s
RU
Response
Conc
M

H

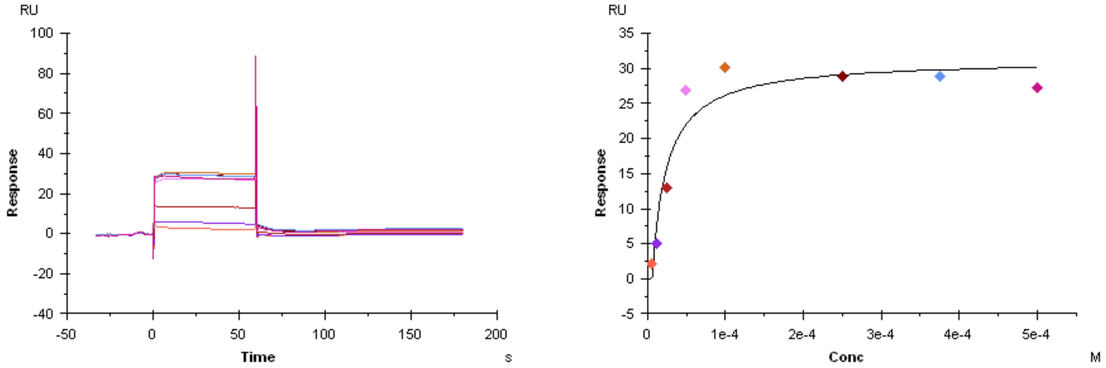
RU
Response
Time
s
RU
Response
Conc
M

I

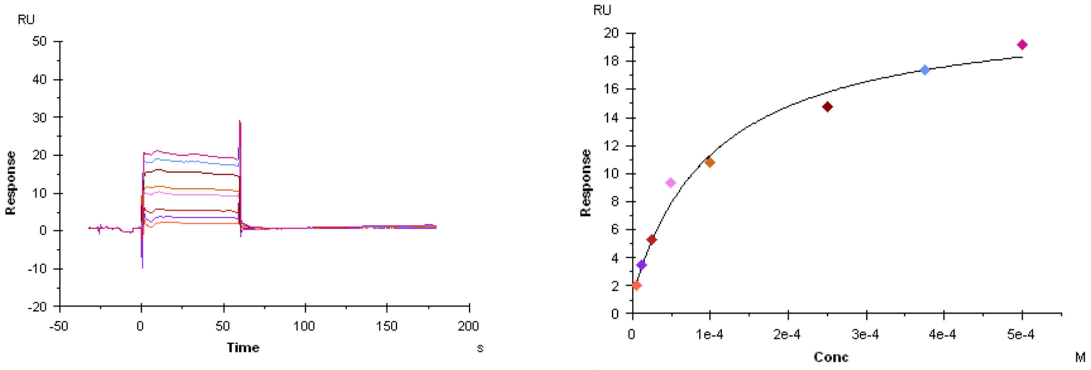
RU
Response
Time
s
RU
Response
Conc
M

J

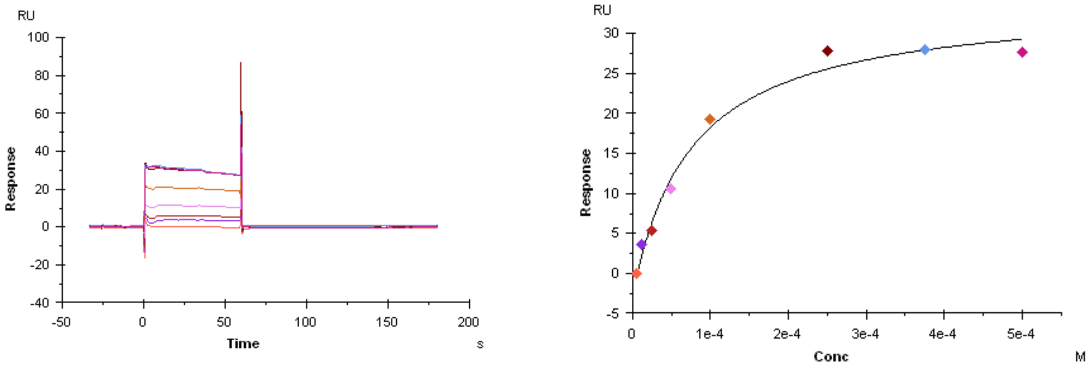
RU
Response
Time
s
RU
Response
Conc
M

Supplementary Figure S7. SPR profiles of vHTS hits binding HSC70-NBD. Representative SPR sensorgrams and Langmuir isotherms for vHTS hits (**a**) **4**, (**b**) **5**, (**c**) **6**, (**d**) **7** and (**e**) **8** binding to WT HSC70-NBD. Representative SPR sensorgrams and Langmuir isotherms for vHTS hits (**f**) **4**, (**g**) **5**, (**h**) **6**, (**i**) **7** and (**j**) **8** binding to S275W HSC70-NBD.

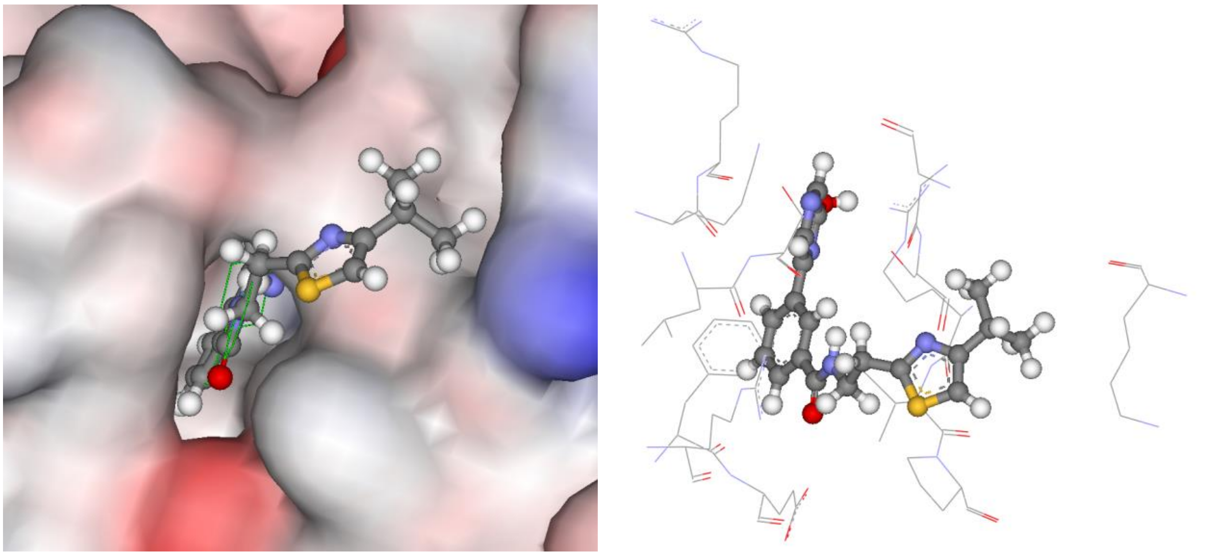

(a) **(b)**

Supplementary Figure S8. The docked pose of compound **4** in the cryptic pocket of HSP70 as predicted by the ASP scoring function. (**a**) Compound **4** shown as ball-and-stick and the crystallized ligand **1** depicted as green lines. The protein surface shown where blue depicts regions with a partial positive charge on the surface, red depicts regions with a partial negative charge and grey shows neutral areas. (**b**) The predicted conformation depicted as ball-and-stick; no specific interactions are predicted between the ligand and the protein. The adjacent amino acids (< 5 Å), buttressing the ligand, are shown as lines. Hydrogen atoms of the amino acids are omitted for clarity.

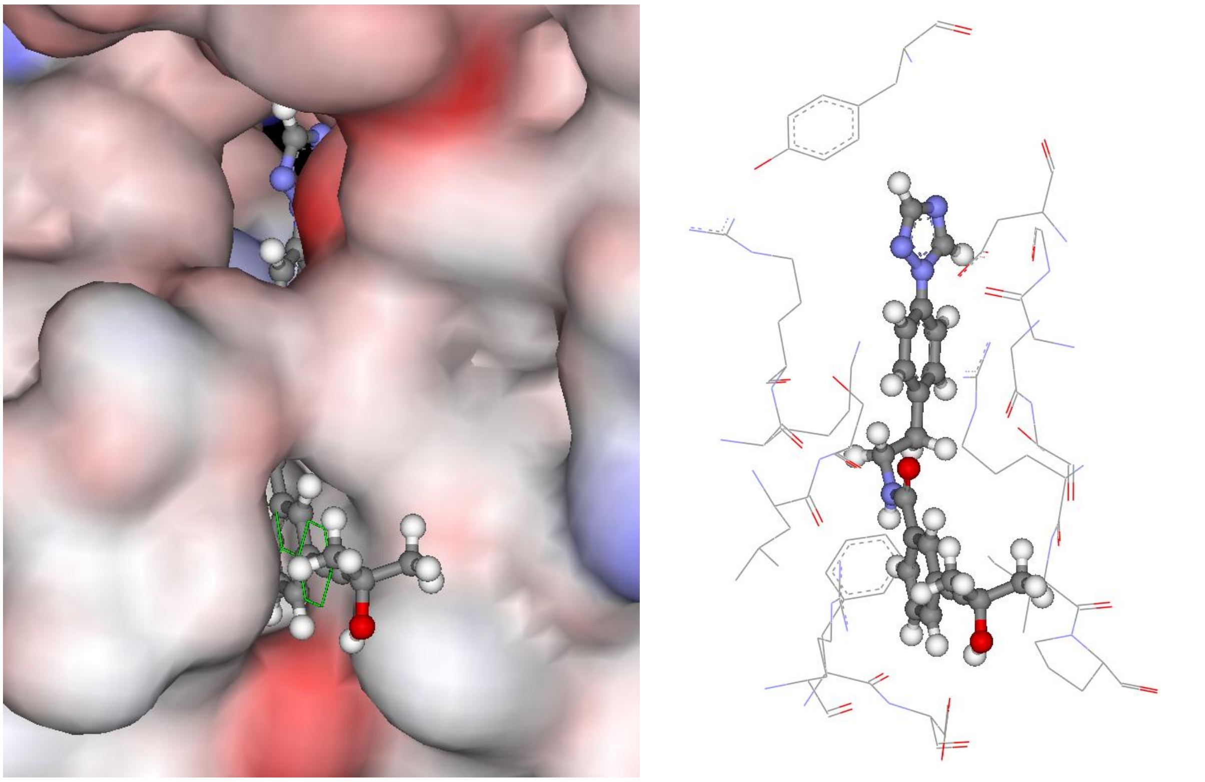

(a) **(b)**

Supplementary Figure S9. The docked pose of compound **5** in the cryptic pocket of HSP70 as predicted by the ASP scoring function. (**a**) Compound **5** shown as ball-and-stick and the crystallized ligand **1** depicted as green lines. The protein surface shown where blue depicts regions with a partial positive charge on the surface, red depicts regions with a partial negative charge, and grey shows neutral areas. (**b**) The predicted conformation depicted as ball-and-stick; no specific interactions are predicted between the ligand and the protein. The adjacent amino acids (< 5 Å), buttressing the ligand, are shown as lines. Hydrogen atoms of the amino acids are omitted for clarity.

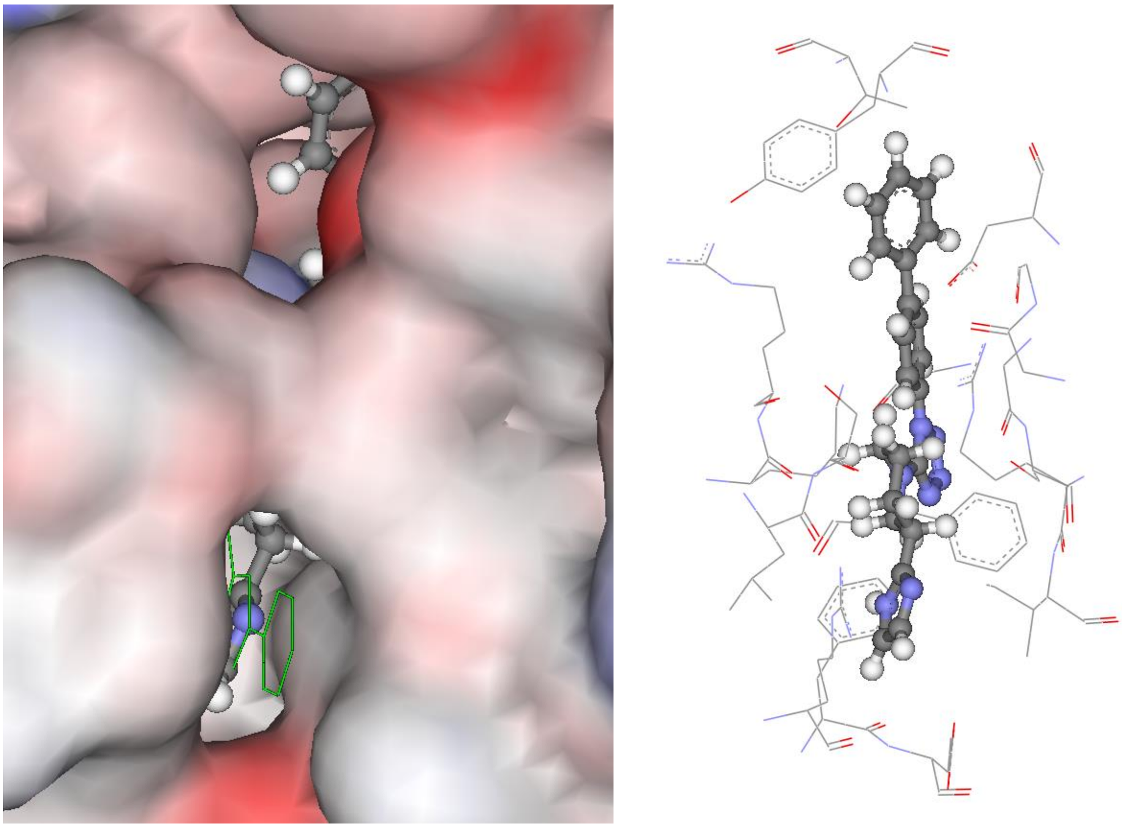

(a) **(b)**

Supplementary Figure S10. The docked pose of compound **6** in the cryptic pocket of HSP70 as predicted by the ASP scoring function. (**a**) Compound **6** shown as ball-and-stick and the crystallized ligand **1** depicted as green lines. The protein surface shown where blue depicts regions with a partial positive charge on the surface, red depicts regions with a partial negative charge, and grey shows neutral areas. (**b**) The predicted conformation depicted as ball-and-stick; no specific interactions are predicted between the ligand and the protein. The adjacent amino acids (< 5 Å), buttressing the ligand, are shown as lines. Hydrogen atoms of the amino acids are omitted for clarity.

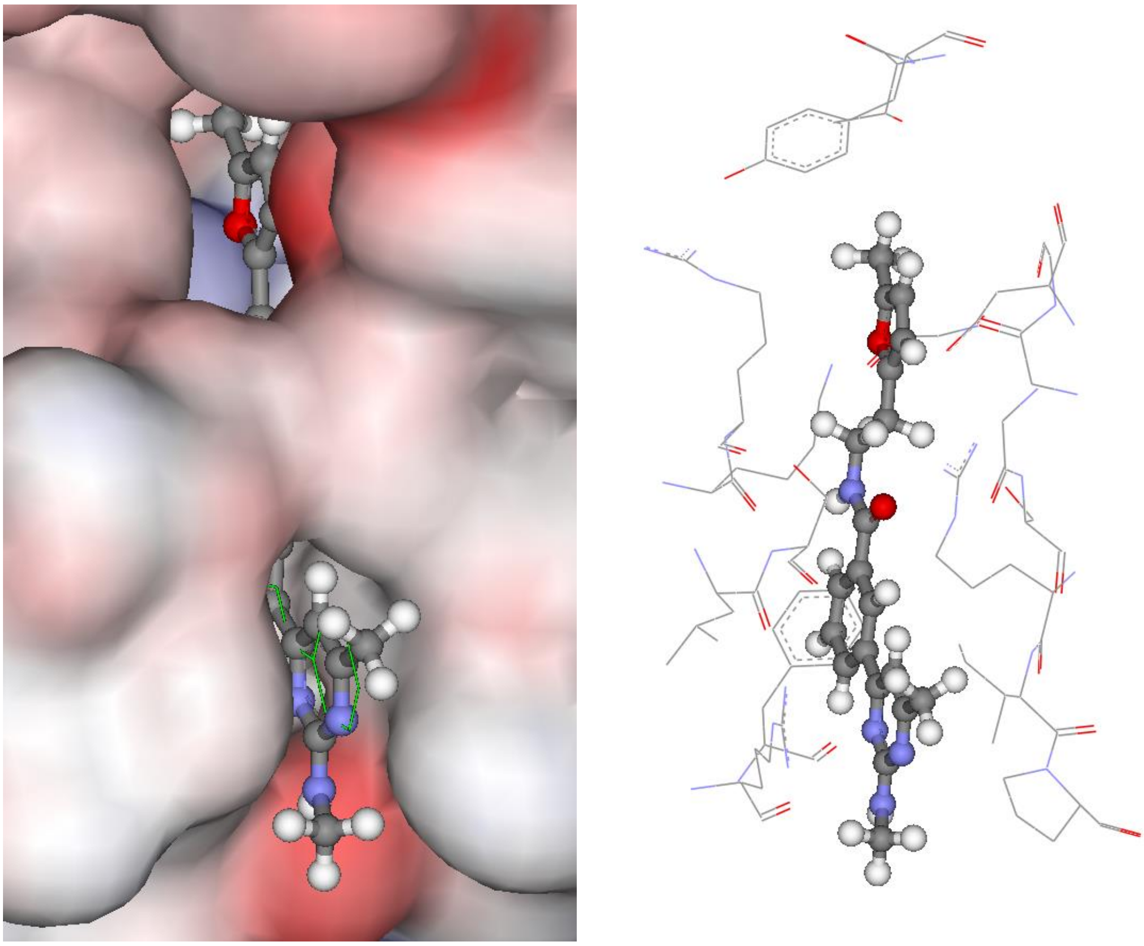

(a) **(b)**

Supplementary Figure S11. The docked pose of compound **7** in the cryptic pocket of HSP70 as predicted by the ASP scoring function. (**a**) Compound **7** shown as ball-and-stick and the crystallized ligand **1** depicted as green lines. The protein surface shown where blue depicts regions with a partial positive charge on the surface, red depicts regions with a partial negative charge, and grey shows neutral areas. (**b**) The predicted conformation depicted as ball-and-stick; no specific interactions are predicted between the ligand and the protein. The adjacent amino acids (< 5 Å), buttressing the ligand, are shown as lines. Hydrogen atoms of the amino acids are omitted for clarity.

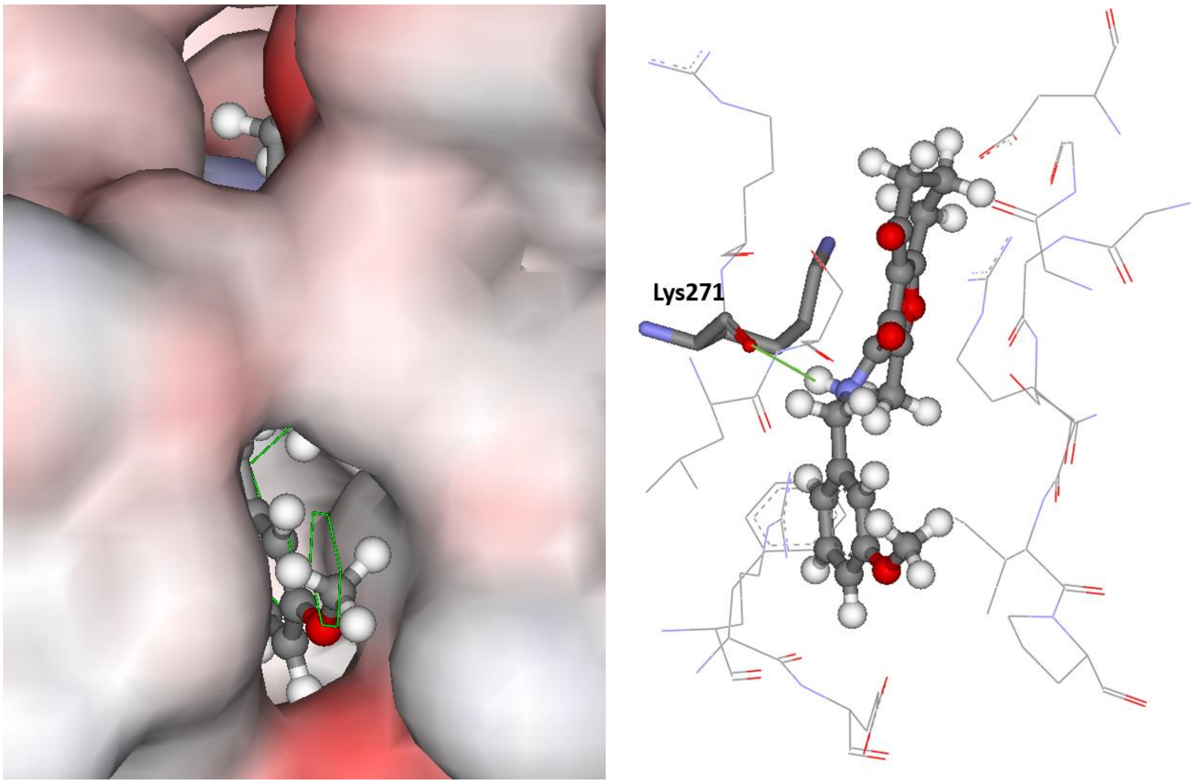

A **B**

Supplementary Figure S12. The docked pose of compound **8** in the cryptic pocket of HSP70 as predicted by the ASP scoring function. (**A**) Compound **8** shown as ball-and-stick format and the crystallized ligand **1** depicted as green lines. The protein surface shown where blue depicts regions with a partial positive charge on the surface, red depicts regions with a partial negative charge, and grey shows neutral areas. (**B**) The predicted conformation depicted as ball-and-stick; hydrogen bonding between the Lys271 carbonyl backbone group and the amine moiety in the ligand is seen (green line). The adjacent amino acids (< 5 Å), buttressing the ligand, are shown as lines. Hydrogen atoms of the amino acids are omitted for clarity.

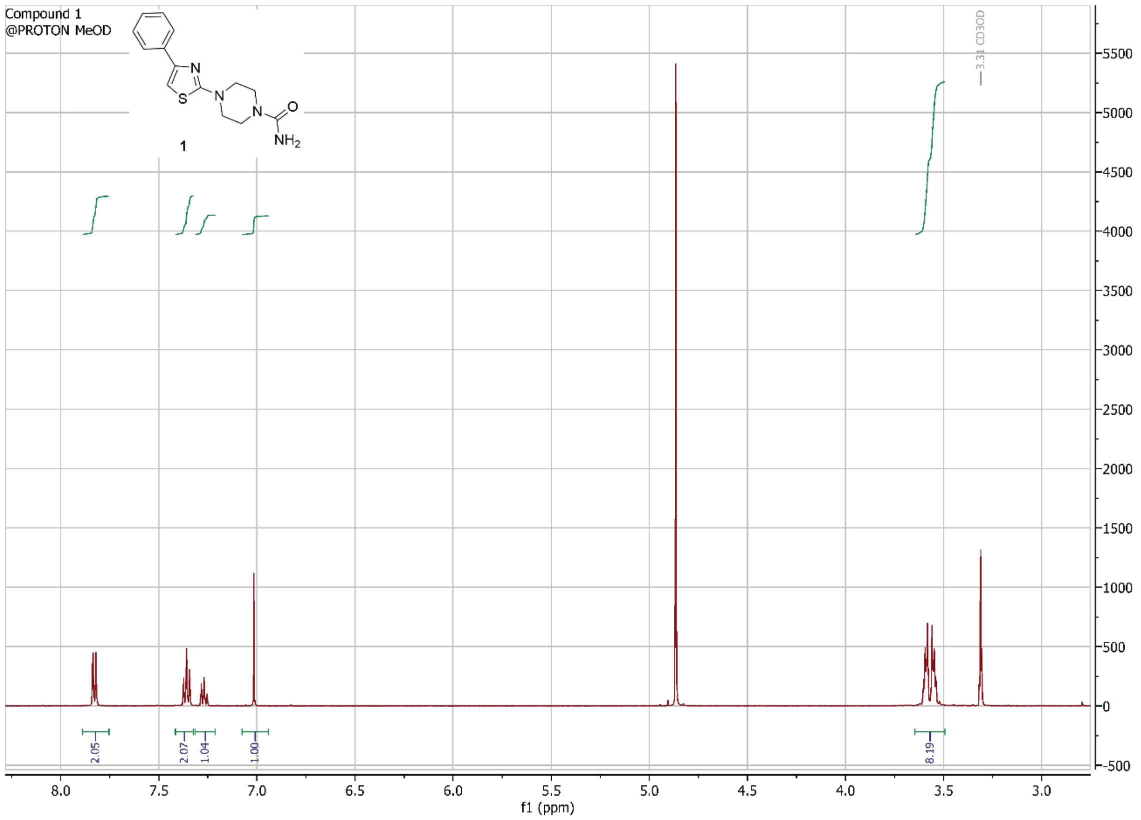
Compound 1
@PROTON MeOD
1
3.31 CD3OD
2.05
2.07
1.04
1.00
8.19
f1 (ppm)

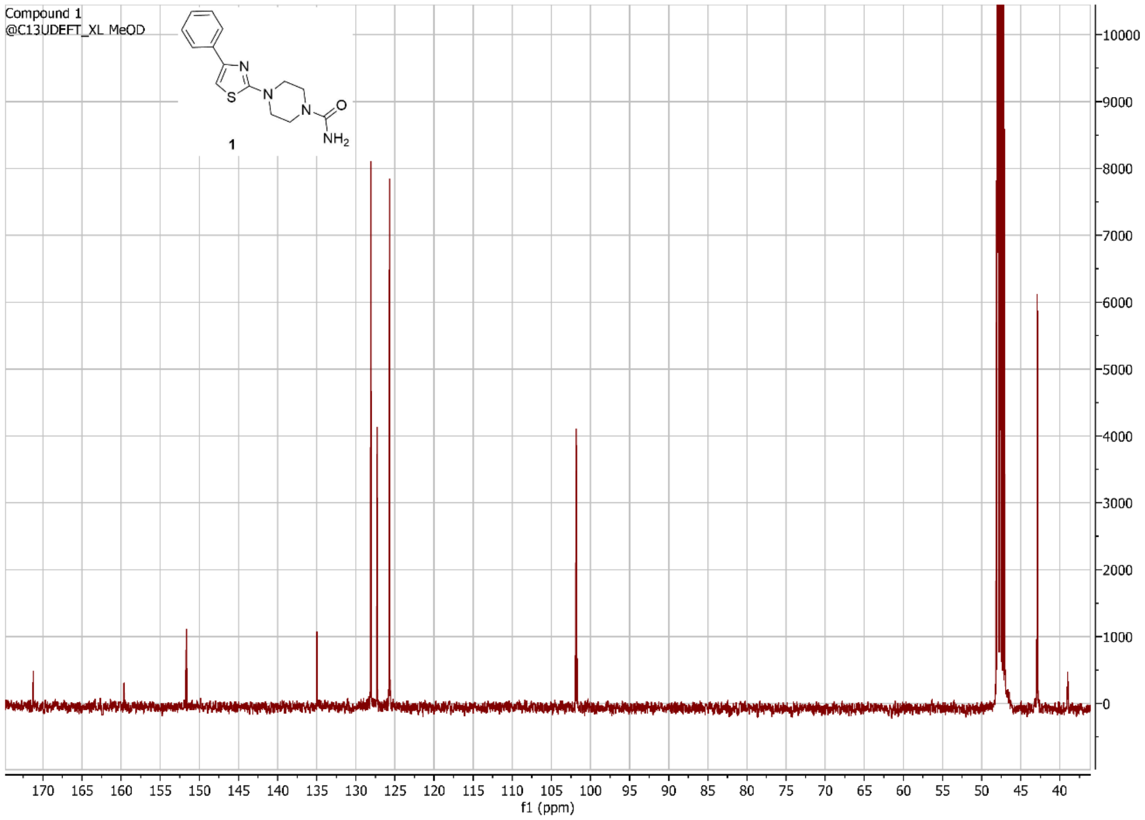
Compound 1
@C13UDEFT_XL MeOD
1
f1 (ppm)

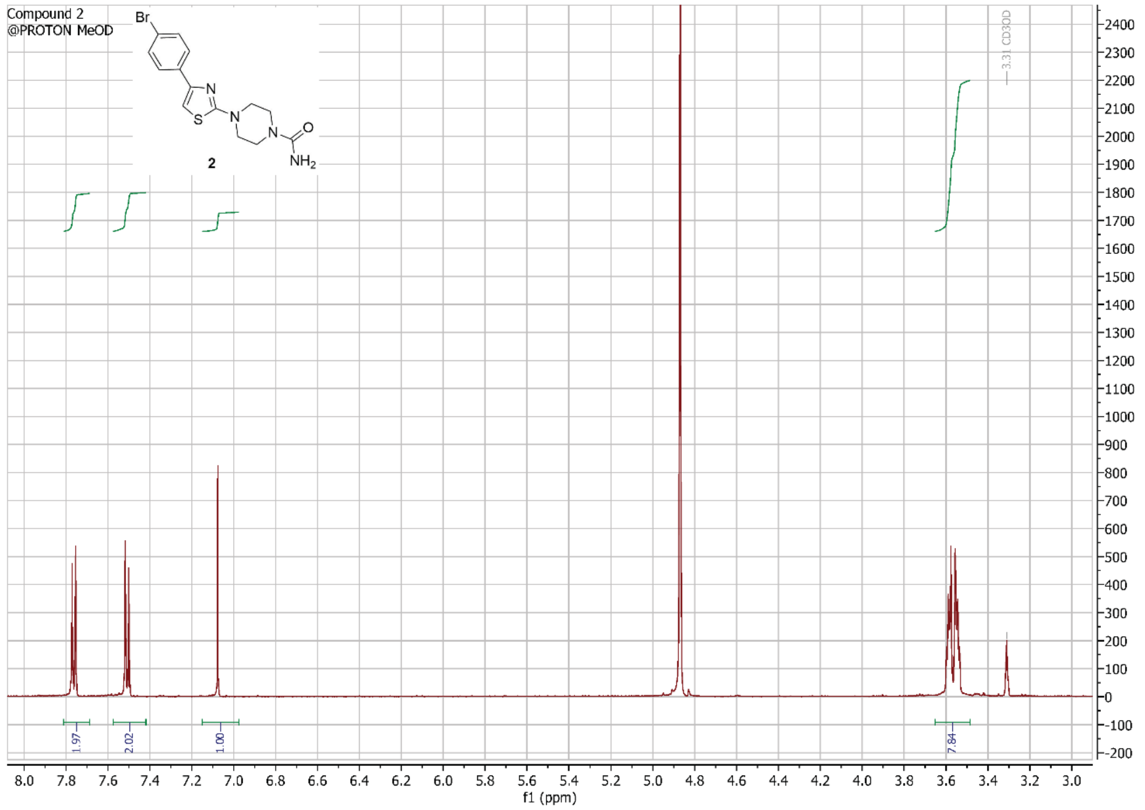

Compound 2
@PROTON MeOD
Br
N
S
N
N
O
NH2
2
3.31 CD3OD
1.97
2.02
1.00
7.84
f1 (ppm)

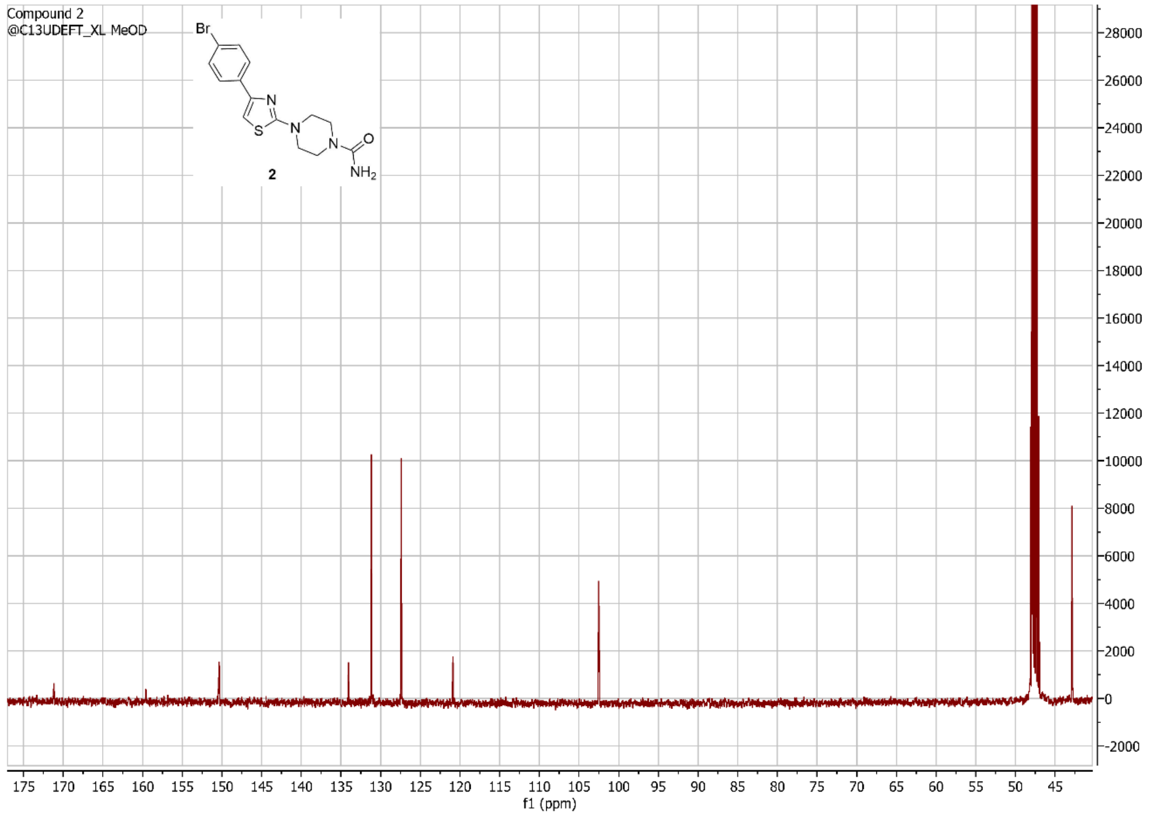

Compound 2
@C13UDEFT_XL MeOD
Br
N
S
N
N
O
NH2
2
f1 (ppm)

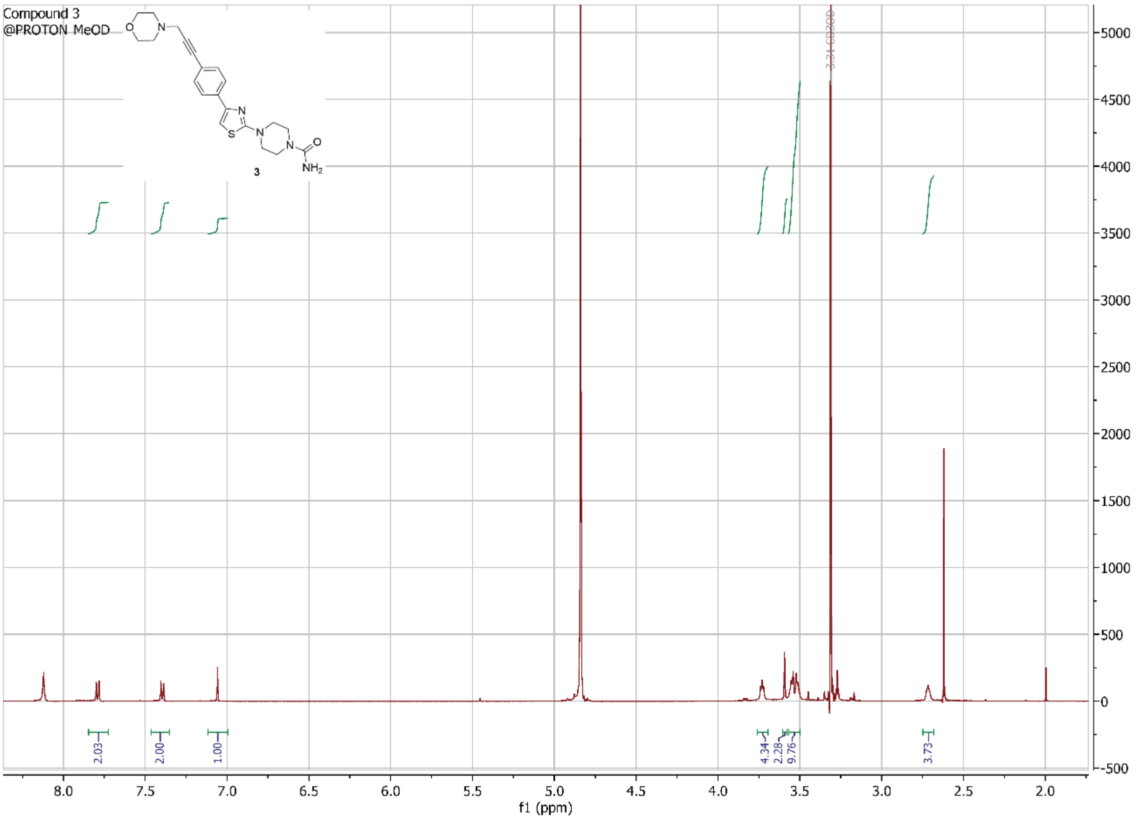

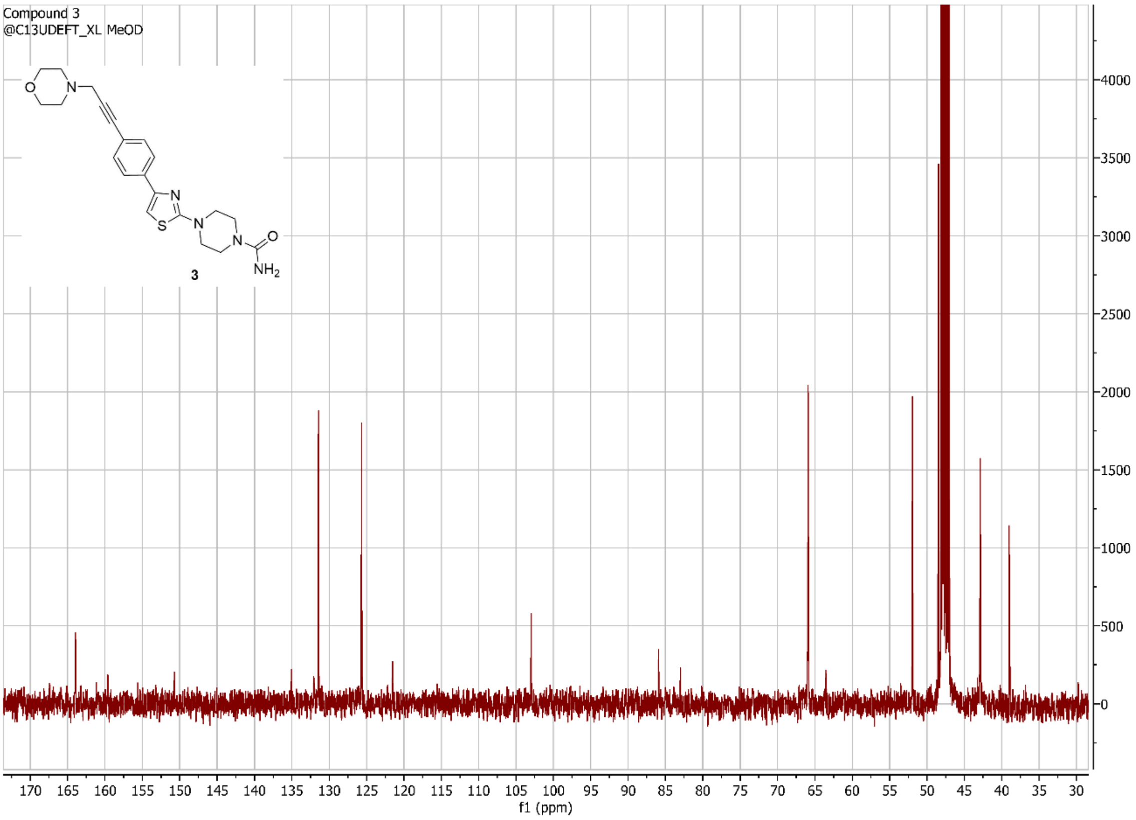

Supplementary Figure S13. ^{1}H and ^{13}C NMR spectra for compounds **1**, **2** and **3**.

Compound **1**

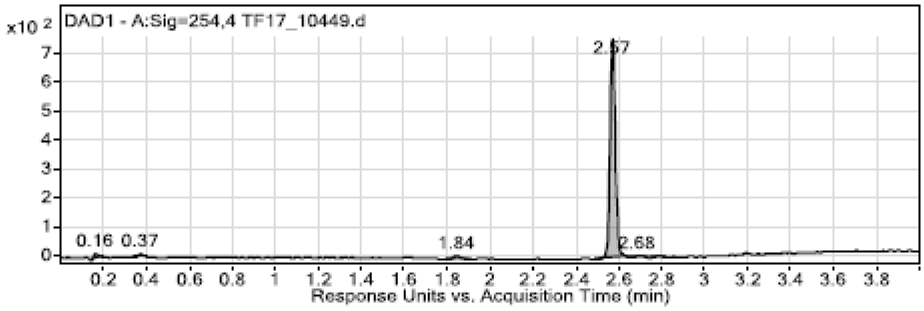

User Chromatogram Peak List

Peak #	RT (mins)	Area	Area Sum %
1	0.16	34.14	2.36
2	0.37	18.78	1.3
3	1.84	34.56	2.39
4	2.57	1328.87	91.85
5	2.68	15.85	1.1
6	2.78	14.65	1.01

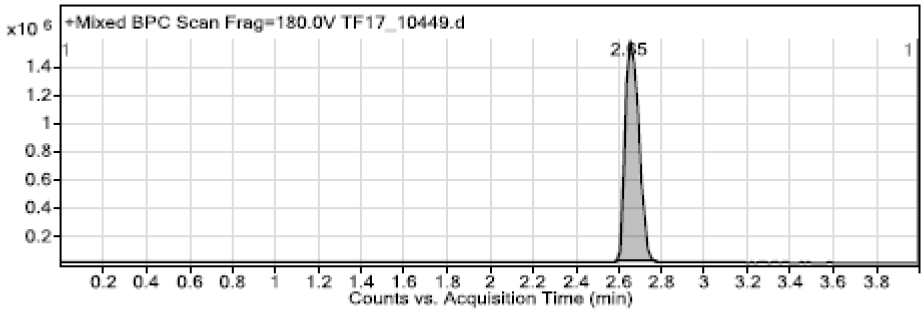

User Chromatogram Peak List

Peak #	RT (mins)	Area	Area Sum %	Base Peak m/z
1	2.65	6945886	100	289.1134

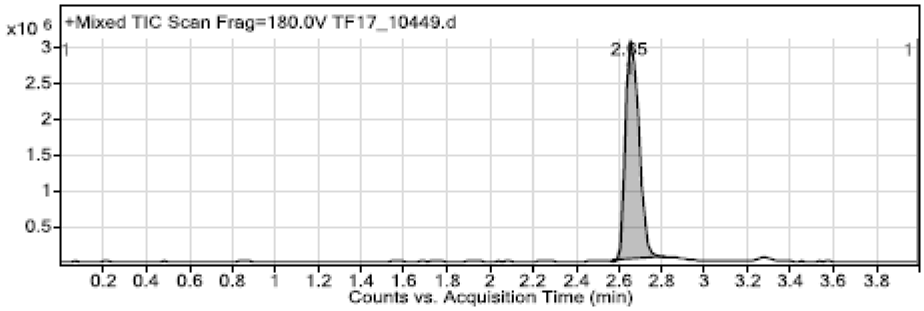

User Chromatogram Peak List

Peak #	RT (mins)	Area	Area Sum %	Base Peak m/z
1	2.65	13459003	100	289.1134

RT
2.65

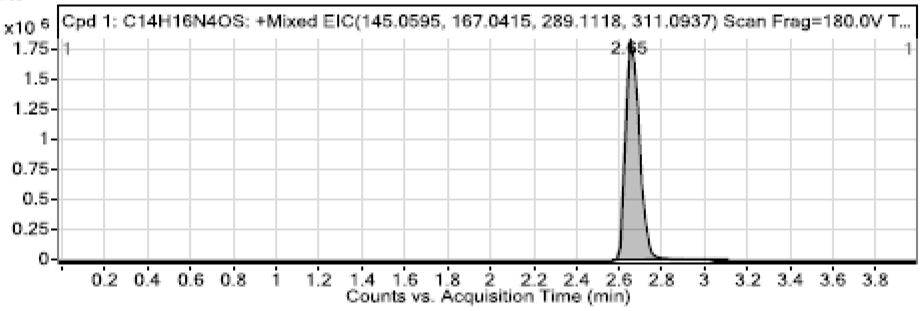

MS Spectrum

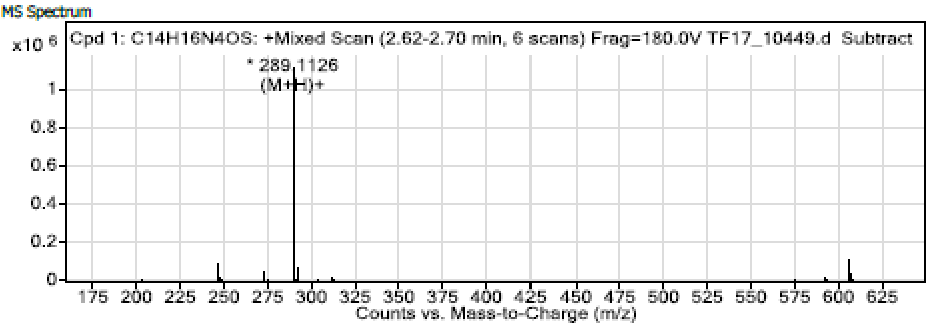

MS Spectrum Peak List

Observed m/z	Calculated Formula [M+H]+	Target m/z	Error (ppm)	Abundance
289.1126	C14 H17 N4 O S	289.1118	3.03	1118560
289.3229				110781
289.4331				6723
289.5367				11208
290.1156	C14 H17 N4 O S	290.1144	3.85	278287
290.3276				13527
291.1124	C14 H17 N4 O S	291.1101	8.03	63929
292.114	C14 H17 N4 O S	292.1116	8.44	9729
311.0936	C14 H16 N4 Na O S	311.0937	-0.43	22912
312.0984	C14 H16 N4 Na O S	312.0964	6.45	5409

--- End Of Report ---

Compound **2**

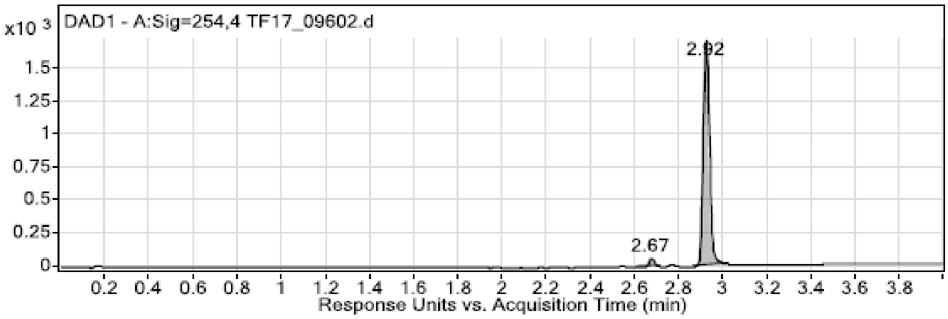

User Chromatogram Peak List

Peak #	RT (mins)	Area	Area Sum %
1	2.67	113.05	3.21
2	2.92	3404.66	96.79

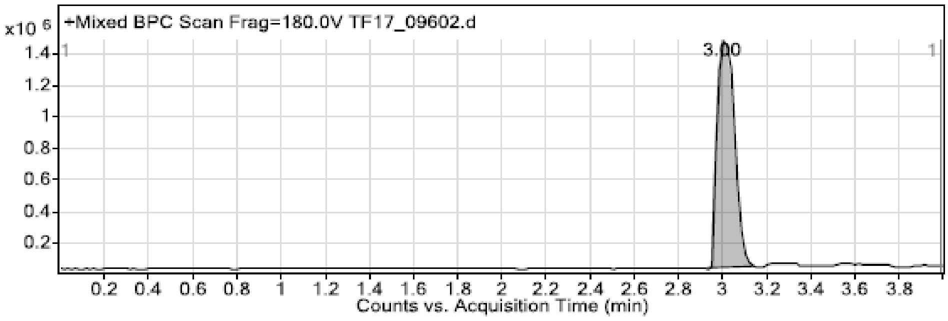

User Chromatogram Peak List

Peak #	RT (mins)	Area	Area Sum %	Base Peak m/z
1	3	7908419	100	369.0167

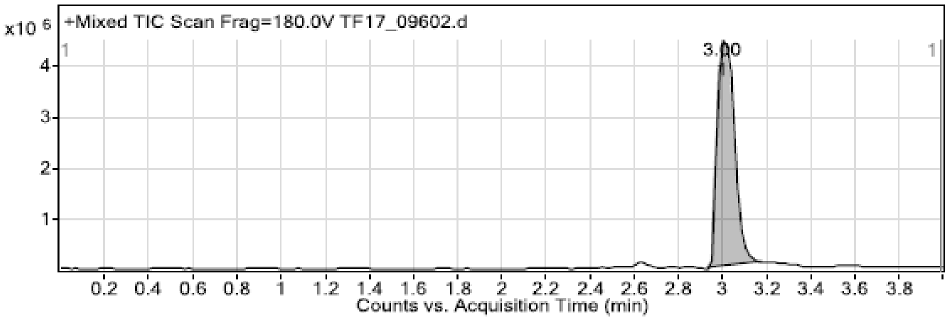

User Chromatogram Peak List

Peak #	RT (mins)	Area	Area Sum %	Base Peak m/z
1	3	24098671	100	369.0167

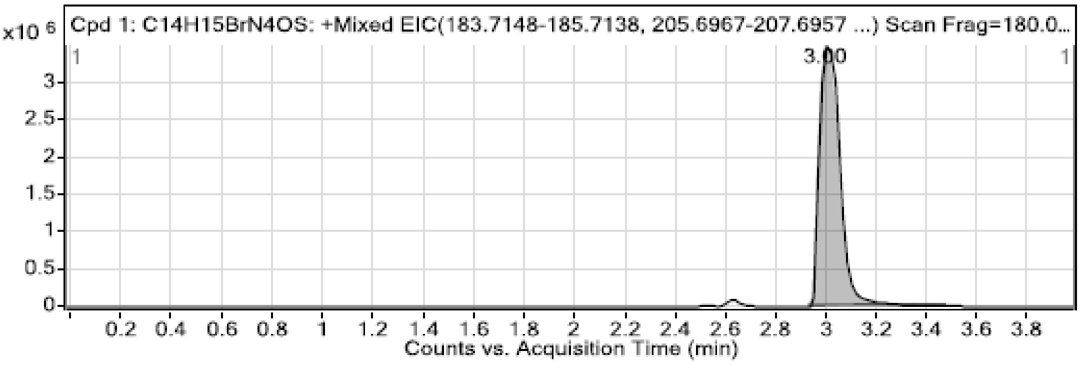

MS Spectrum

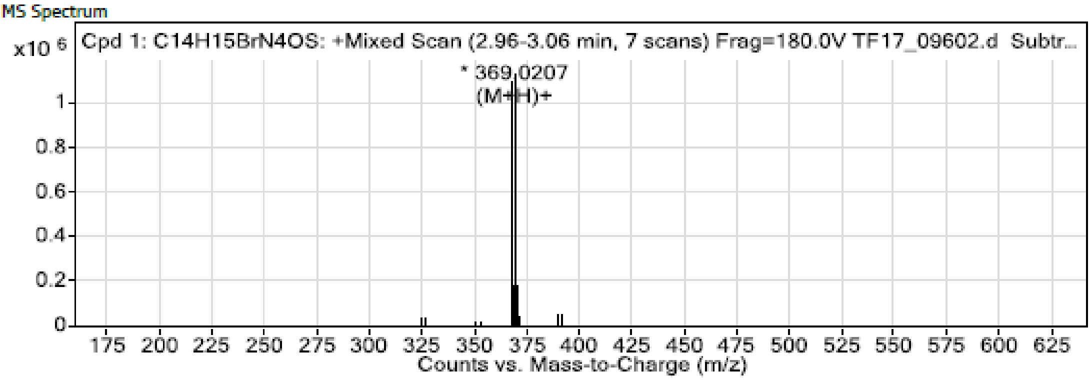

MS Spectrum Peak List

Observed m/z	Calculated Formula [M+H]+	Target m/z	Error (ppm)	Abundance
367.0225	C14 H16 Br N4 O S	367.0223	0.73	1089692
367.2595				94116
368.0245	C14 H16 Br N4 O S	368.025	-1.19	182806
369.0207	C14 H16 Br N4 O S	369.0202	1.15	1127631
369.257				112090
369.4974				13494
370.0225	C14 H16 Br N4 O S	370.0229	-0.89	181124
371.0186	C14 H16 Br N4 O S	371.0186	-0.14	49207
389.004	C14 H15 Br N4 Na O S	389.0042	-0.59	54675
391.0016	C14 H15 Br N4 Na O S	391.0022	-1.64	57026

--- End Of Report ---

Compound **3**

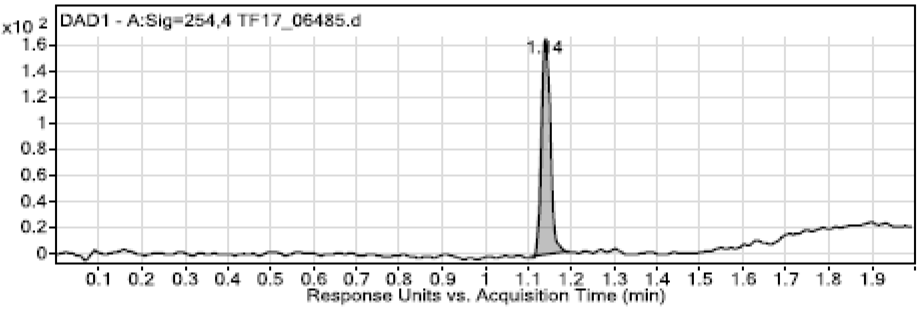

User Chromatogram Peak List

Peak #	RT (mins)	Area	Area Sum %
1	1.14	241.5	100

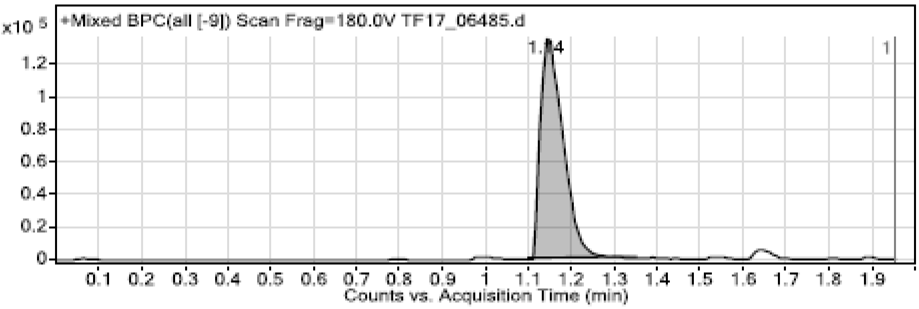

User Chromatogram Peak List

Peak #	RT (mins)	Area	Area Sum %	Base Peak m/z
1	1.14	502236	100	412.178

RT
1.15

MFE MS Spectrum

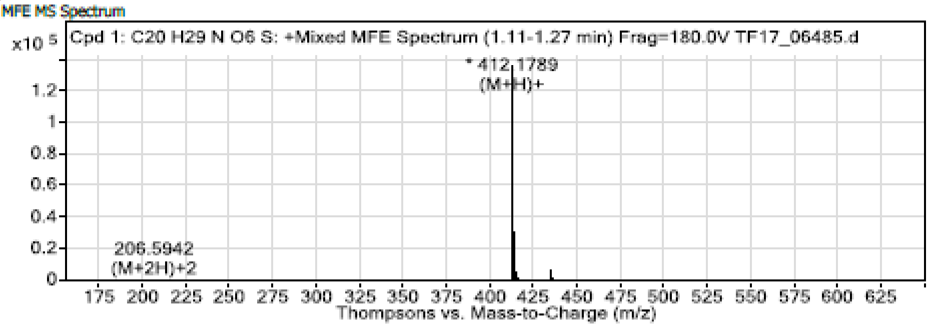

--- End Of Report ---

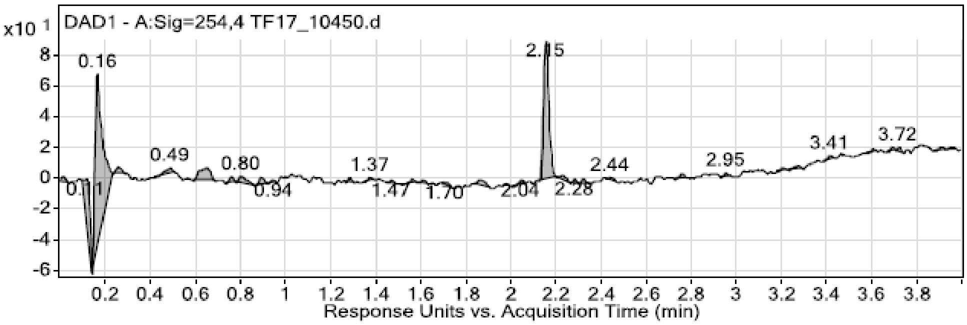

User Chromatogram Peak List

Peak #	RT (mins)	Area	Area Sum %
2	0.11	42.11	6.72
3	0.16	268.64	42.85
4	0.26	7.43	1.18
5	0.49	10.57	1.69
6	0.65	29.58	4.72
7	0.76	5.35	0.85
8	0.8	12.25	1.95
9	0.89	8.66	1.38
17	1.86	8.75	1.4
19	2.15	147.26	23.49

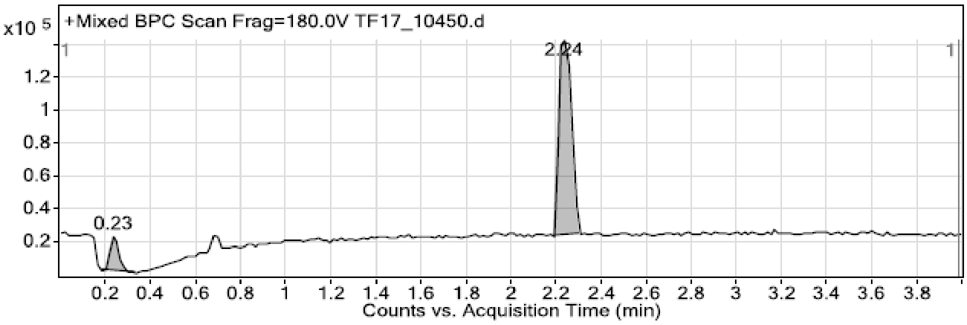

User Chromatogram Peak List

Peak #	RT (mins)	Area	Area Sum %	Base Peak m/z
1	0.23	62265	11.84	274.1086
2	2.24	463460	88.16	412.1801

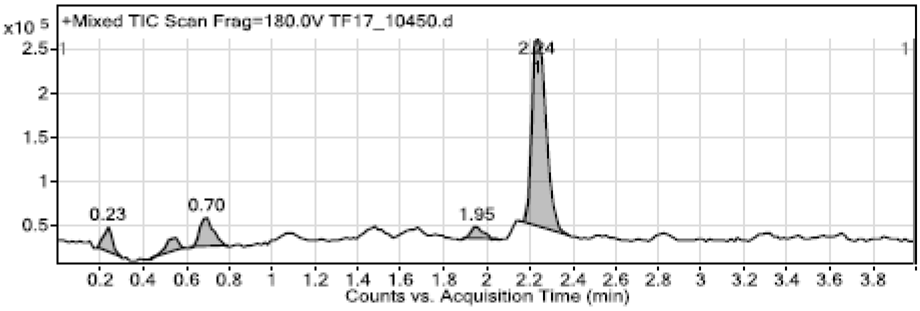

User Chromatogram Peak List

Peak #	RT (mins)	Area	Area Sum %	Base Peak m/z
1	0.23	85211	6.25	274.1086
2	0.55	81660	5.99	195.0891
3	0.7	153066	11.23	306.154
4	1.95	60551	4.44	195.0892
5	2.24	982115	72.08	412.1801

RT
2.24

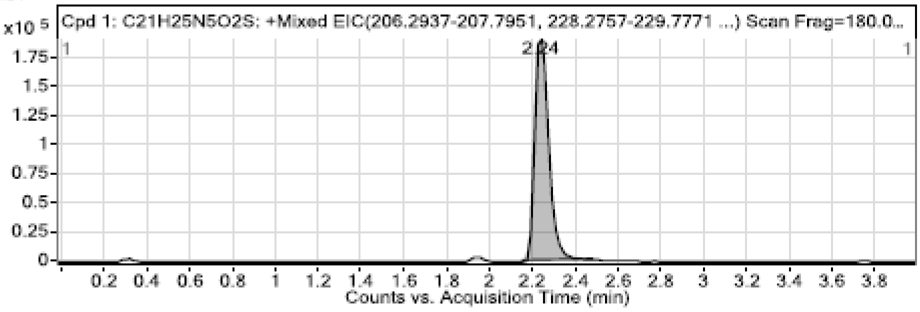

MS Spectrum

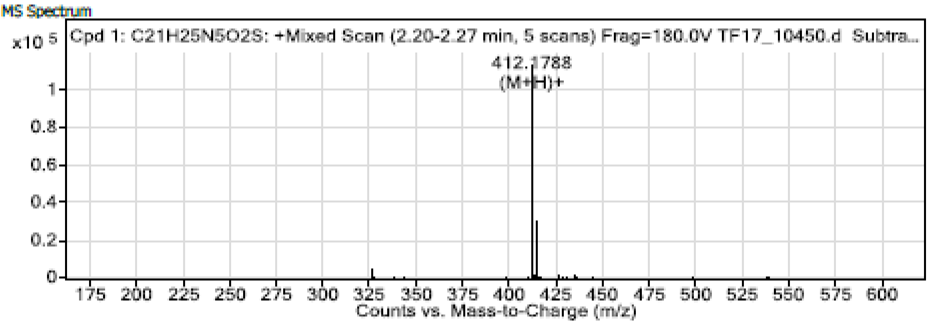

MS Spectrum Peak List

Observed m/z	Calculated Formula [M+H]+	Target m/z	Error (ppm)	Abundance
410.164				1090
411.1669				479
412.1788	C21 H26 N5 O2 S	412.1802	-3.21	113857
412.4315				2137
413.1831	C21 H26 N5 O2 S	413.183	0.29	30286
413.4381				548
414.182	C21 H26 N5 O2 S	414.1803	4.08	7243
415.1834	C21 H26 N5 O2 S	415.1811	5.34	1465
434.1612	C21 H25 N5 Na O2 S	434.1621	-2.04	2307
435.1642	C21 H25 N5 Na O2 S	435.1649	-1.71	741

--- End Of Report ---

Compound **4**

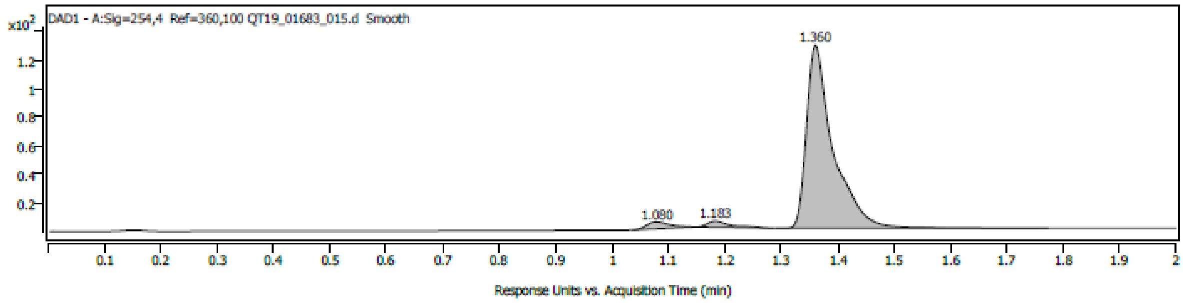

RT (mins)	Area	Area Sum %	Base Peak m/z
1.080	14	3	
1.183	13	3	
1.360	422	94	

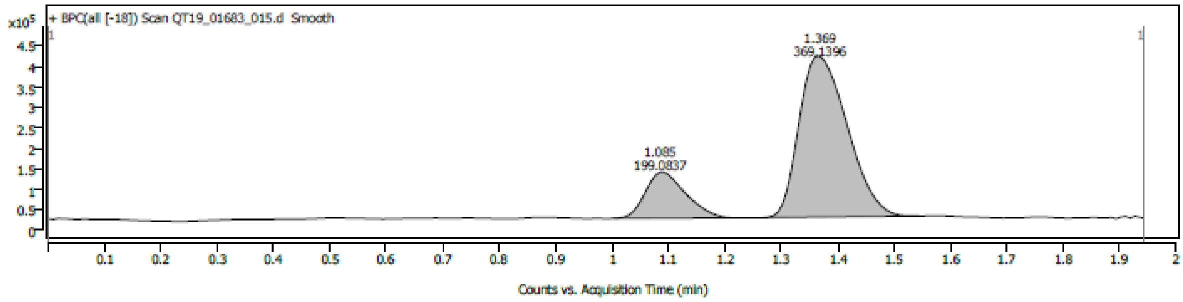

RT (mins)	Area	Area Sum %	Base Peak m/z
1.085	558313	19	199.0837
1.369	2341247	81	369.1396

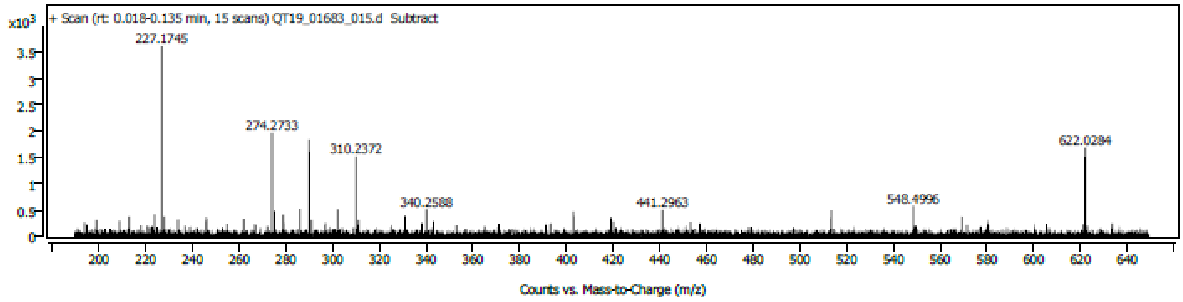

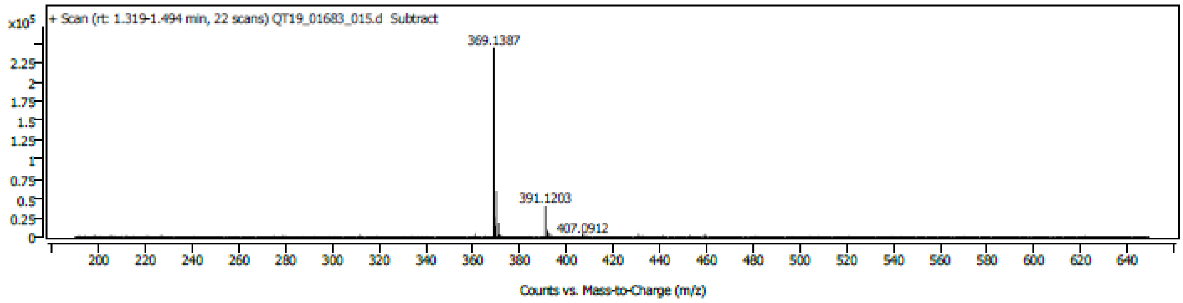

Compound **5**

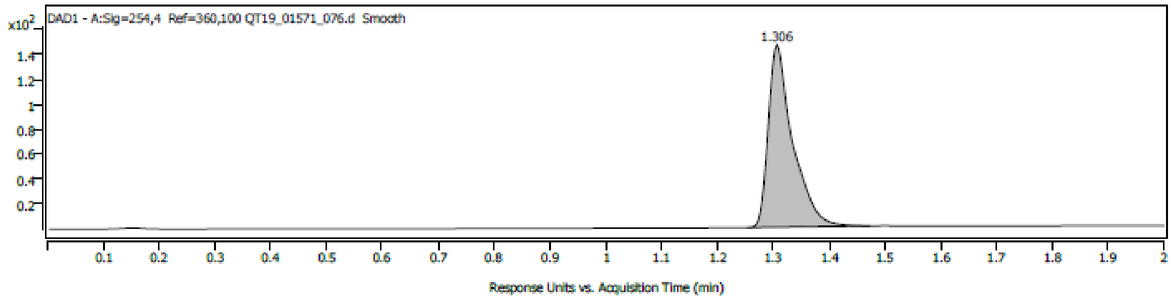

RT (mins)	Area	Area Sum %	Base Peak m/z
1.306	443	100	

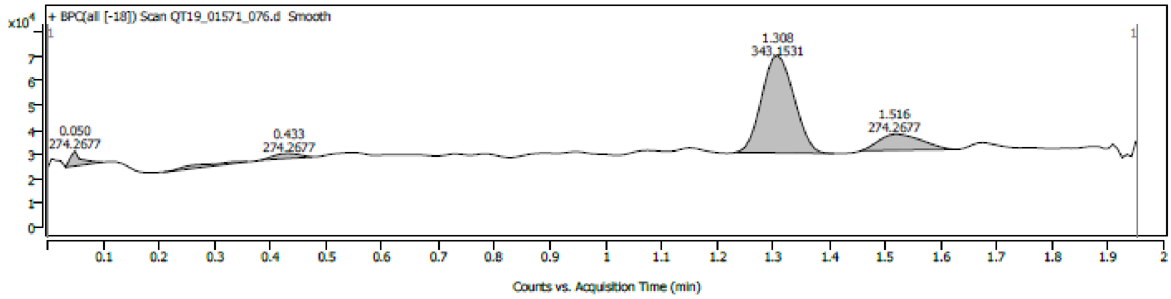

RT (mins)	Area	Area Sum %	Base Peak m/z
0.050	8936	4	274.2677
0.341	7866	4	274.2677
0.433	6870	3	274.2677
1.308	161195	73	343.1531
1.516	35220	16	274.2677

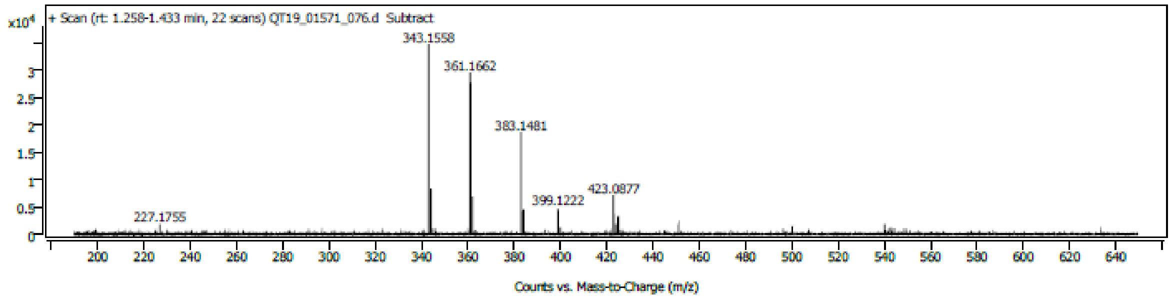

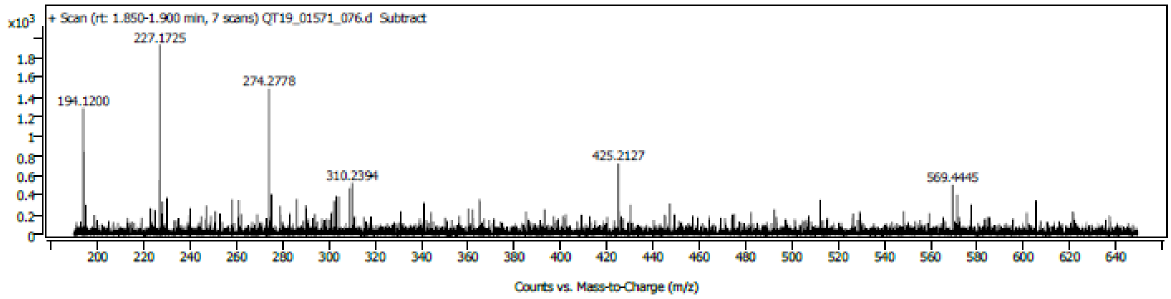

Compound **6**

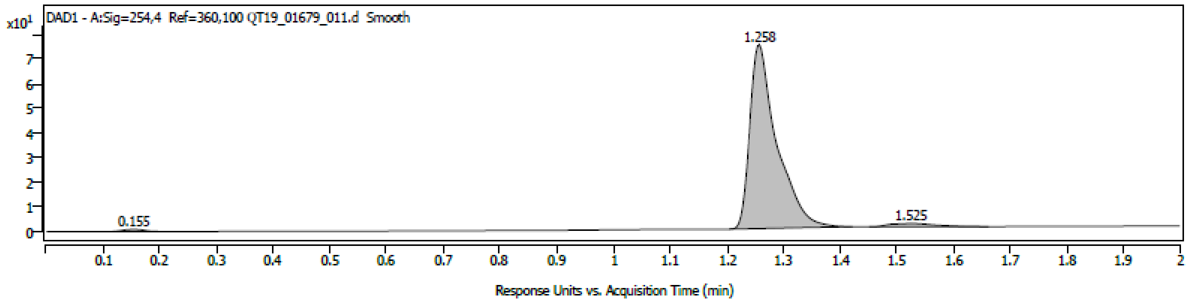

RT (mins)	Area	Area Sum %	Base Peak m/z
0.155	3	1	
1.258	249	96	
1.525	7	3	

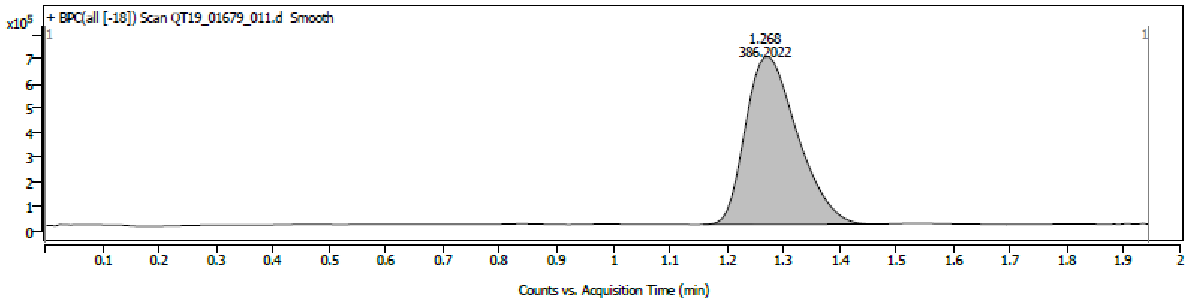

RT (mins)	Area	Area Sum %	Base Peak m/z
1.268	4350384	100	386.2022

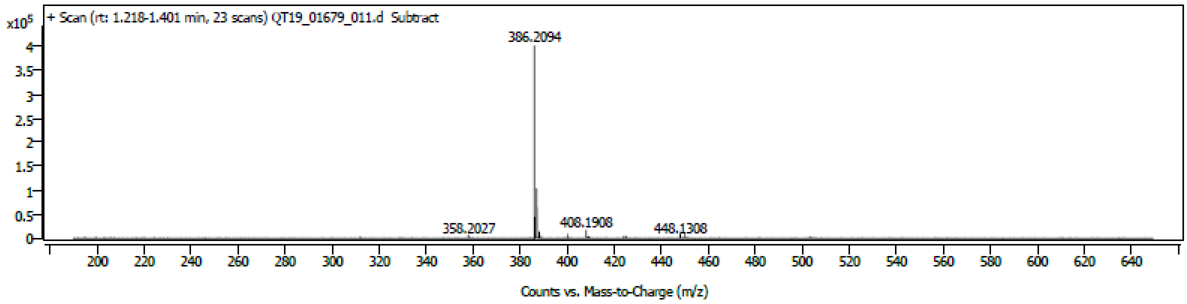

Compound **7**

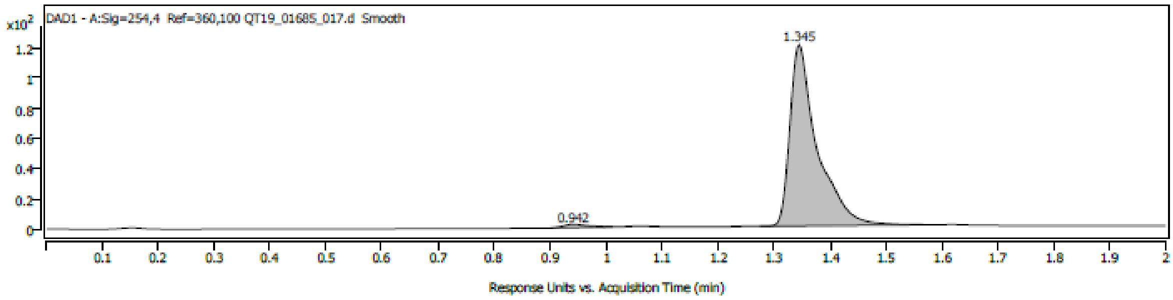

RT (mins)	Area	Area Sum %	Base Peak m/z
0.942	6	2	
1.345	405	98	

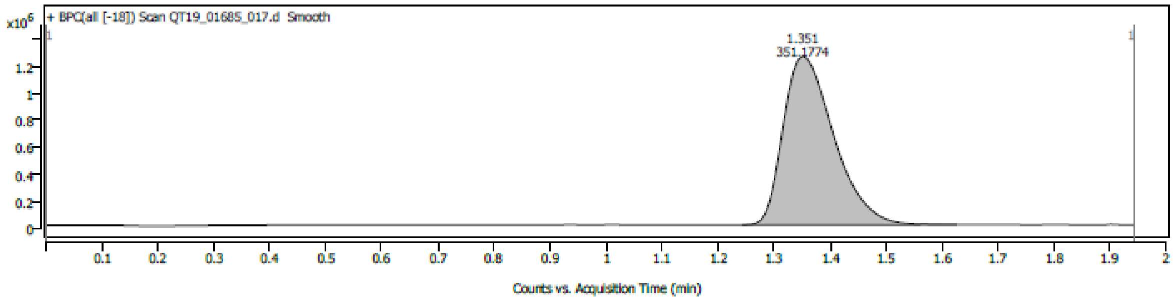

RT (mins)	Area	Area Sum %	Base Peak m/z
1.351	7785943	100	351.1774

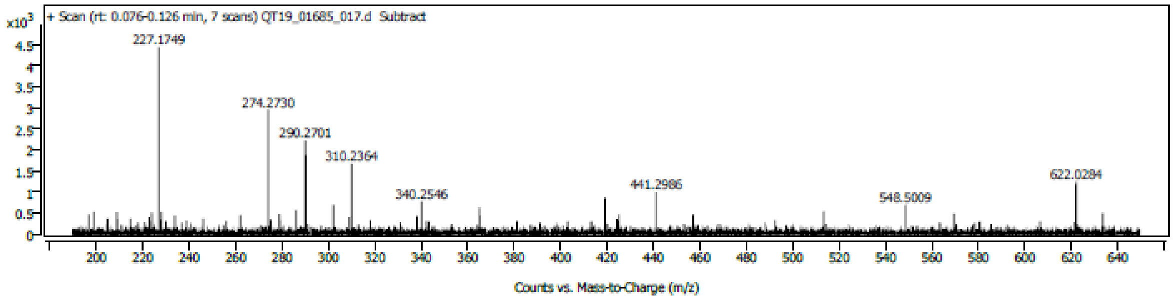

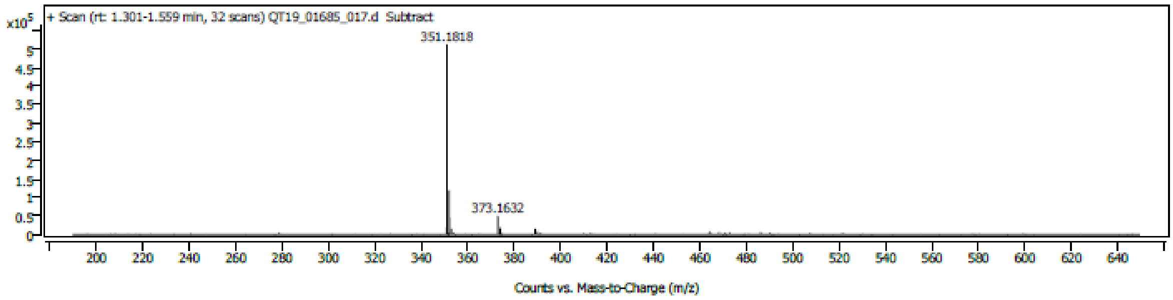

Compound **8**

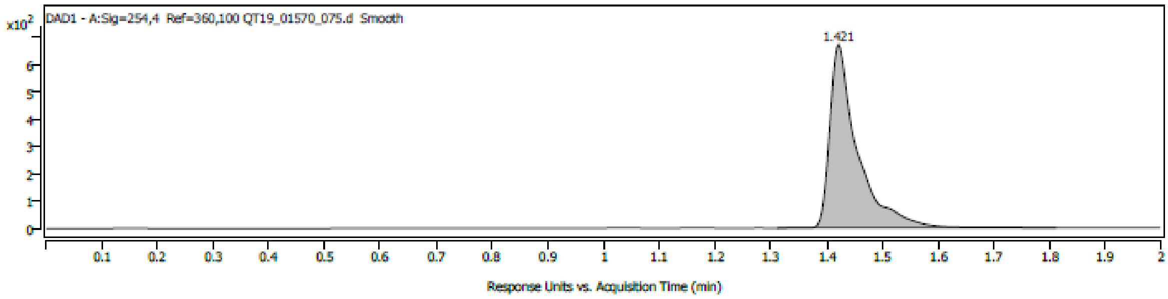

RT (mins)	Area	Area Sum %	Base Peak m/z
1.421	2324	100	

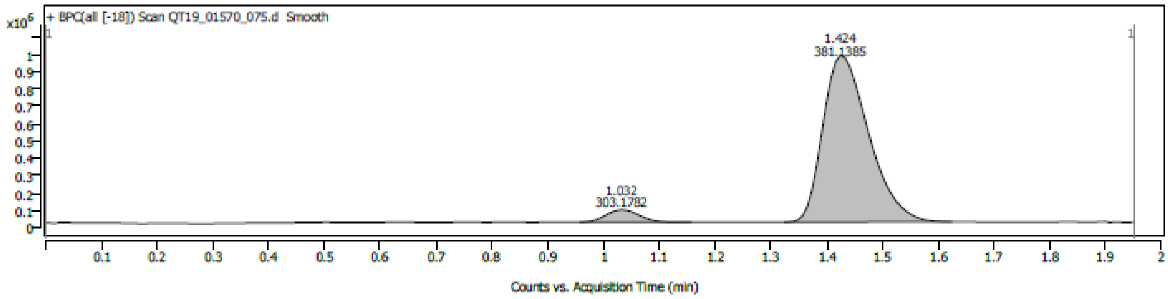

RT (mins)	Area	Area Sum %	Base Peak m/z
1.032	293845	5	303.1782
1.424	5480366	95	381.1385

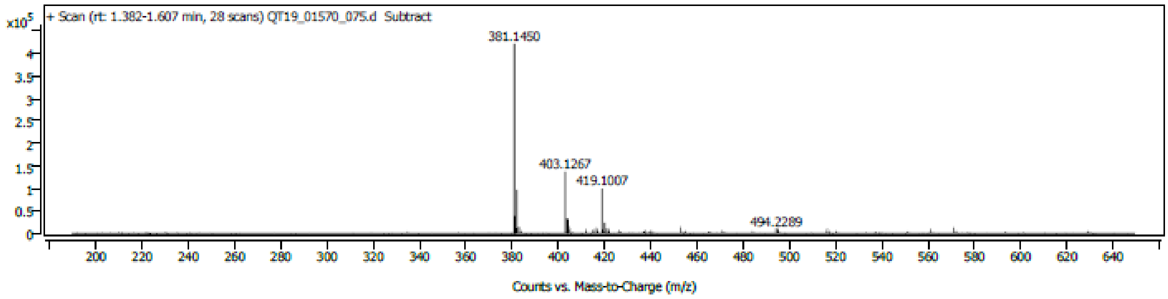

Supplementary Figure S14: LC-MS spectra for compounds **1 – 8**.

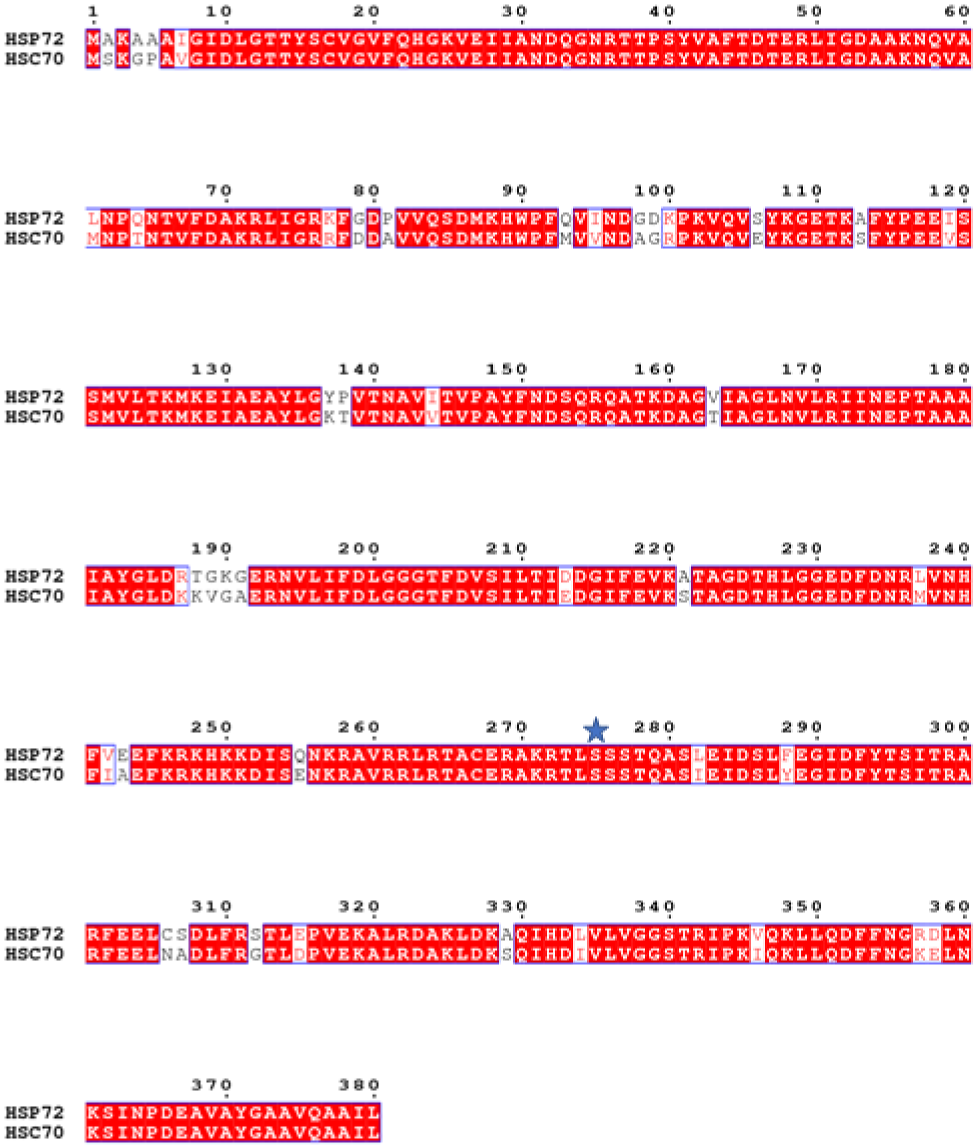

Supplementary Figure S15: Sequence alignment of the nucleotide binding domains of human HSP72 and HSC70 proteins. Identical residues are shown in white font on a red background and similar residues in red font on a white background. Residue S275, crucial for the binding of the adenine ring of ATP, is highlighted with a blue star. Sequence identity is 85.1 % on the full-length proteins, rising to 88.7 % for the nucleotide binding domains only (residues 1 to 380 of both proteins), and to 100 % for the residues coating the ATP binding site and/or the binding site of compound **1** (residues 201-204, 229-231, 267-276, 299-303, 339-345 and 366). The sequence alignment was performed with Clustal Omega and its visual representation with ESpript 3.0.

Multiplet	NN	Range	Abs. Integral	Concentration	SNR
A(d)	2	7.81..7.72	6942472.5126	297.2180	1012.0886
B(t)	2	7.44..7.38	6939013.8658	297.0699	1076.0009
C(t)	1	7.37..7.31	3259444.4027	279.0837	525.9891

Concentration Average: 291.1239 uM

RMSD%: 2.9245

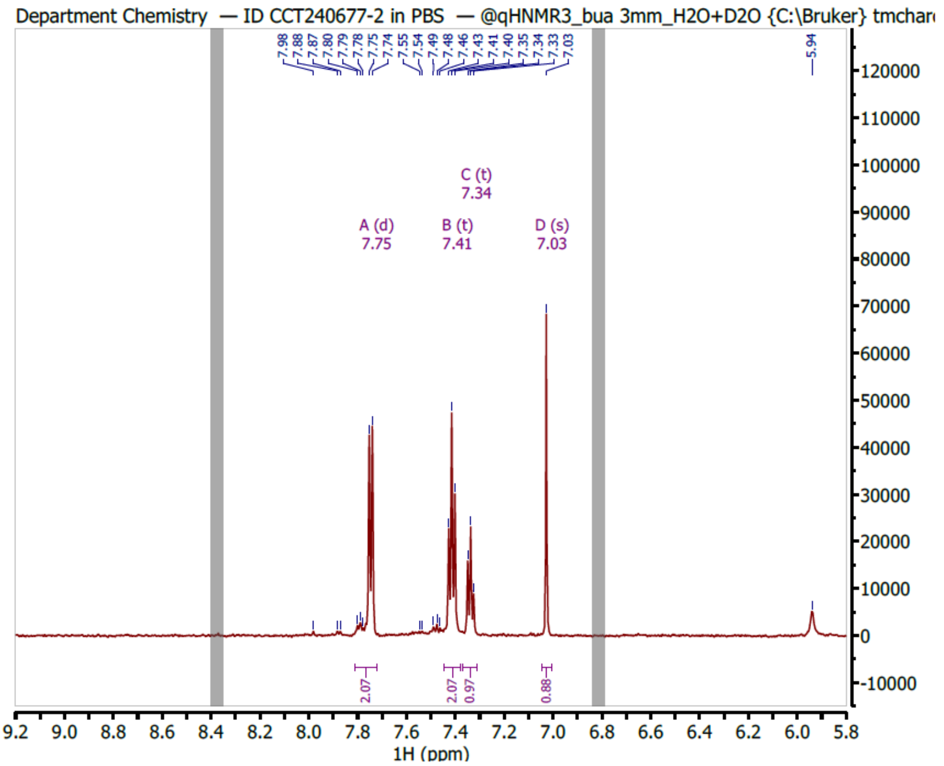

Parameter	Value
Title	TFFM-5208-005-CCT240677-2.10.fid
Solvent	3mm_H2O+D2O
Temperature	298.0
Pulse Sequence	lc1pngppsf2
Probe	Z75812_0002 (CP TCI 600S3 H-C/ N-D-05 Z)
Number of Scans	32
Receiver Gain	101.0
Relaxation Delay	20.0000
Acquisition Date	2019-06-22T15:12:21
Spectrometer Frequency	600.14

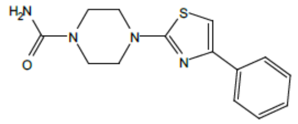

Supplementary Figure S16: Determination of pH 7.4 phosphate buffer solubility of **1** by quantitative NMR.

Supplementary Table S1. Observed changes in NMR signal intensities for **3** on addition of HSP72-NBD and ATP.[1]

Conditions	Peak a	Peak b	Peak c
**3** + HSP72-NBD	41%	36%	31%
**3** + HSP72-NBD + ATP	5%	22%	7%

[1] Quantified from the data shown in Supplementary Figure S4 using MestreNova

Supplementary Table S2. LC-MS data to confirm the identity and purity of vHTS hits **4 – 8**.

Compound	Mass detected	Purity (UV)
**4**	369.1 $(M+H)^+$	94%
**5**	361.2 $(M+H)^+$	100%
**6**	386.2 $(M+H)^+$	96%
**7**	351.2 $(M+H)^+$	98%
**8**	381.1 $(M+H)^+$	100%

Supplementary Table S3: Crystallographic data collection and refinement statistics for **1**-HSP72-NBD.

Protein construct	HSP72 1-380
Ligand	**1**
PDB code	7Q4R
Crystal	
Space group	$P\ 2_1\ 2_1\ 2_1$
Unit cell dimensions (a/b/c in Å)	47.47/78.47/93.50
Unit cell angles (α/β/γ in °)	90/90/90
Data collection and processing	
Beamline	DLS I24
Wavelength (Å)	0.9686
Integration program	XDS
Reduction program	AIMLESS
Resolution range	47.47 – 1.79
Number of unique reflections [a]	32590 (1470)
Completeness [a]	96.8 (75)
Redundancy [a]	5.5 (3.4)
R_{merge} (%) [a]	5.9 (37.7)
I/σ(I) [a]	15.7 (2.2)
$CC_{1/2}$ [a, b]	0.999 (0.864)
Refinement	
Program	BUSTER
R_{work} (%)	19.17
R_{free} (%)	23.49
Number of residues	383
Number of water molecules	409
Average B-factor (Å^2)	26.30
Ramachandran favoured (%)	100
Ramachandran outliers (%)	0
RMSD bonds (Å)	0.012
RMSD angles (°)	1.496

[a] Values in parentheses are for the highest resolution shell; [b] Half-dataset correlation coefficient [1].

Reference

1. Karplus, P.A.; Diederichs, K. Linking crystallographic model and data quality. *Science* **2012**, *336*, 1030–1033. https://doi.org/10.1126/science.1218231.
